# Protein-aggregating ability of different protoporphyrin-IX nanostructures is dependent on their oxidation and protein-binding capacity

**DOI:** 10.1016/j.jbc.2021.100778

**Published:** 2021-05-21

**Authors:** Dhiman Maitra, Benjamin M. Pinsky, Amenah Soherawardy, Haiyan Zheng, Ruma Banerjee, M. Bishr Omary

**Affiliations:** 1 Center for Advanced Biotechnology and Medicine, Rutgers University, Piscataway, New Jersey, USA; 2 University of Michigan Medical School, Ann Arbor, Michigan, USA; 3 Department of Biological Chemistry, Ann Arbor, Michigan, USA

**Keywords:** porphyrin nanostructure, porphyria, protein aggregation, oxidative stress, photodynamic therapy, BSA, bovine serum albumin, Copro, coproporphyrin, DMA, N,N-dimethylacetamide, ^1^O_2_, singlet oxygen, PP-IX, Protoporphyrin-IX, Φ, quantum yield, ROS, reactive oxygen species, UPLC, ultra performance liquid chromatography, Uro, uroporphyrin, UV-Vis, ultraviolet–visual

## Abstract

Porphyrias are rare blood disorders caused by genetic defects in the heme biosynthetic pathway and are associated with the accumulation of high levels of porphyrins that become cytotoxic. Porphyrins, due to their amphipathic nature, spontaneously associate into different nanostructures, but very little is known about the cytotoxic effects of these porphyrin nanostructures. Previously, we demonstrated the unique ability of fluorescent biological porphyrins, including protoporphyrin-IX (PP-IX), to cause organelle-selective protein aggregation, which we posited to be a major mechanism by which fluorescent porphyrins exerts their cytotoxic effect. Herein, we tested the hypothesis that PP-IX-mediated protein aggregation is modulated by different PP-IX nanostructures *via* a mechanism that depends on their oxidizing potential and protein-binding ability. UV–visible spectrophotometry showed pH-mediated reversible transformations of PP-IX nanostructures. Biochemical analysis showed that PP-IX nanostructure size modulated PP-IX-induced protein oxidation and protein aggregation. Furthermore, albumin, the most abundant serum protein, preferentially binds PP-IX dimers and enhances their oxidizing ability. PP-IX binding quenched albumin intrinsic fluorescence and oxidized His-91 residue to Asn/Asp, likely *via* a previously described photo-oxidation mechanism for other proteins. Extracellular albumin protected from intracellular porphyrinogenic stress and protein aggregation by acting as a PP-IX sponge. This work highlights the importance of PP-IX nanostructures in the context of porphyrias and offers insights into potential novel therapeutic approaches.

Protoporphyrin-IX (PP-IX) is nature’s template for several essential biomolecules including heme, chlorophyll, coenzyme F430 (in methanogenic bacteria), and vitamin B12 (1). The PP-IX macrocycle consists of four pyrrole rings connected by methene bridges and two ionizable propioniate side chains. The highly conjugated macrocycle with 18 π-electrons confers PP-IX with distinct UV-visible absorbance and fluorescence properties (2). Due to its amphiphilic nature, PP-IX tends to aggregate in aqueous solution (3). Indeed, PP-IX has been reported to exist as detergent micellarized 0.56 kDa monomers (4), dimers (5), tetramers (6), and higher-order structures that are >70 kDa in size (4). The spontaneous self-association of PP-IX occurs by a combination of hydrogen bonding (between the propionate carboxylate) and π-π stacking of the porphine ring (3, 6, 7). The resulting face-to-face or “H-aggregates” have the propionate chains of adjacent PP-IX molecules in a “head-to-tail” orientation (3, 6, 7). These supramolecular higher-order structures offer advantages over their constituent monomers (8, 9). The self-assembling property of PP-IX and similar compounds are being explored for a variety of applications (10, 11, 12), *e.g.*, synthetic porphyrins for photodynamic therapy (13, 14, 15), photovoltaic cells (16, 17, 18), photocatalysis (19, 20, 21, 22, 23), and sensor applications (23, 24, 25). Although porphyrin macromolecular species have been extensively studied, their role in the context of porphyria and in the context of protein aggregation is not known.

Porphyrias include eight genetic disorders that are caused by mutations in any of the eight enzymes in the heme biosynthetic pathway (26, 27). Heme biosynthesis starts in the mitochondrion where aminolevulinic acid (ALA) synthase catalyzes the rate-limiting conversion of glycine and succinyl Co-A to δ-ALA (27, 28, 29, 30). Upon translocation to the cytosol, ALA is converted in several steps to the first cyclic tetrapyrrole, uroporphyrinogen, which is then converted to coproporphyrinogen (27, 28, 29, 30). Coproporphyrinogen then enters the mitochondria, where it is converted to heme *via* formation of protoporphyrinogen and PP-IX (27, 28, 29, 30). Porphyrinogens are colorless, nonfluorescent (31) compounds, which are auto-oxidized to more stable and fluorescent porphyrins (27). Porphyrins are toxic metabolites, and their levels are tightly regulated. In porphyrias, blockages in the heme biosynthetic pathway due to enzyme mutations lead to the accumulation of intermediates with consequent organ and tissue damage (4, 18, 25).

Porphyrin-mediated tissue damage is proposed to occur through reactive oxygen species (ROS) generated by type I/II photosensitized reactions of porphyrins (32, 33, 34). However, the precise nature of the ROS as well as the specific targets of porphyrin-generated ROS is poorly understood (27). Accumulating findings have demonstrated the unique properties of fluorescent porphyrins to cause organelle-selective protein aggregation though a mechanism that involves a “porphyrination-deporphyrination” cycle (27, 35, 36, 37, 38, 39). In this cycle, PP-IX binds to target proteins (porphyrination) and induces localized unfolding and conformational change (40, 41). Photosensitization of protein-bound PP-IX generates singlet oxygen (^1^O_2_), then oxidation of specific methionines to methionine sulfone or sulfoxide (27, 42). Subsequent noncovalent interactions lead to protein-PP-IX lattice-like aggregate formation. During deporphyrination, acidic pH or high-salt conditions lead to release of PP-IX and disaggregation of the proteins (27, 42). We posit that this proteotoxic property of porphyrins is a major mechanism for tissue damage in porphyrias that involve fluorescent porphyrin accumulation.

Herein, we tested the hypothesis that PP-IX-mediated protein aggregation is modulated by PP-IX speciation into selective supramolecular structures that lead to differential oxidizing potential and protein binding.

## Results

### pH-mediated spontaneous and reversible transformations of PP-IX nanostructures

**Figure 1:**
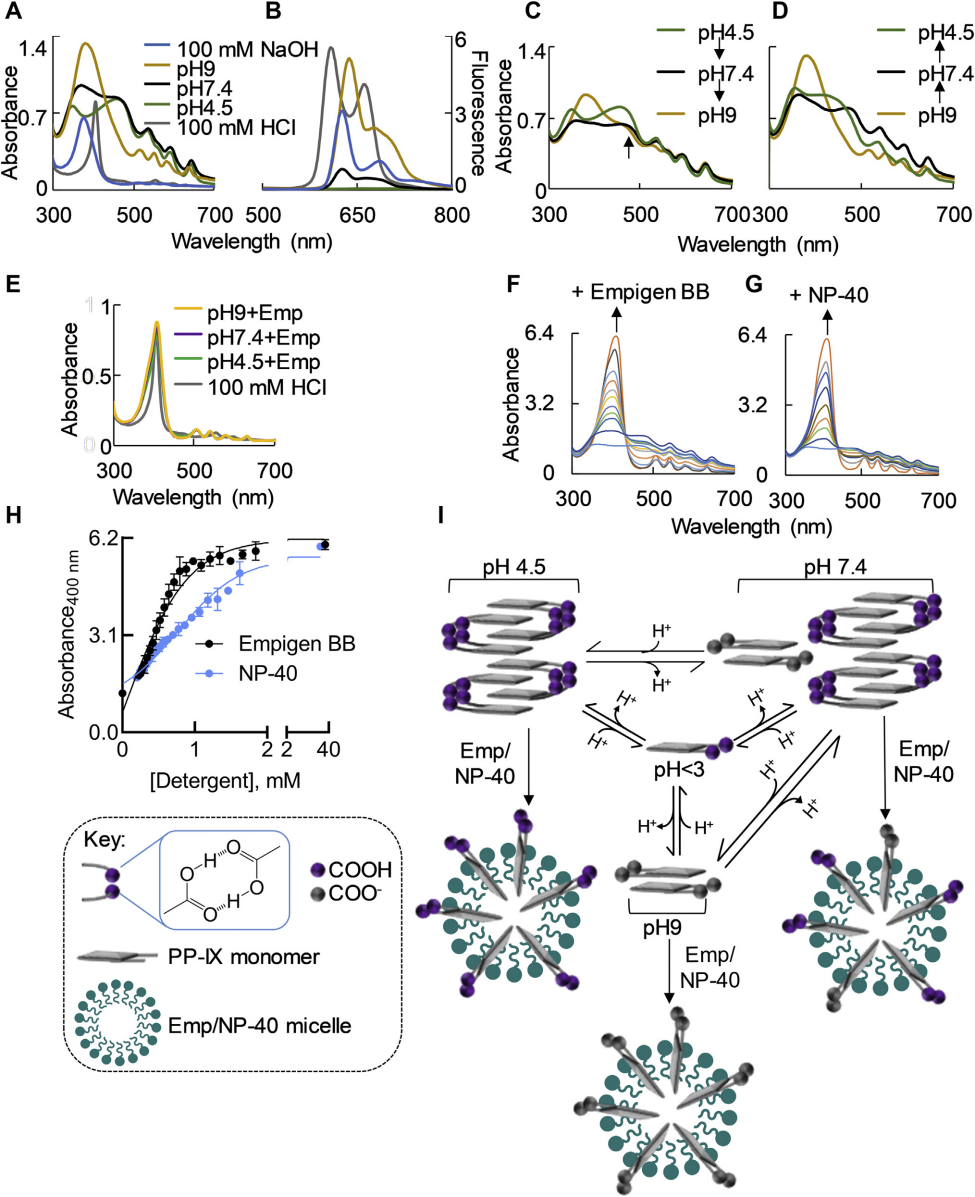
**pH-mediated spontaneous and reversible transformations of PP-IX nanostructures**. *A*, absorbance spectra of PP-IX (50 μM) in pH 4.5, 7.4, 9; and PP-IX (10 μM) in 100 mM NaOH/HCl. *B*, the PP-IX solutions from *panel A* were excited at 375 nm followed by collection of the fluorescence emission spectra (500–800 nm). *C*, PP-IX stock solution was diluted in pH 4.5 buffer to a final concentration of 50 μM. The resulting solution was divided into two aliquots, and the pH was then adjusted to 7.4 or 9 using 5 N NaOH. Absorbance spectra of the resulting pH 9 and pH 7.4 solutions were recorded and compared with the absorbance spectra of PP-IX solution at pH 4.5. *D*, PP-IX stock solution was diluted using pH 9 buffer to a final concentration of 50 μM. The resulting solution was divided into two aliquots, and the pH was adjusted to 7.4 and 4.5 using 5 N HCl. Absorbance spectra of the resulting pH 7.4 and pH 4.5 solutions were recorded and compared with the absorbance spectra of PP-IX solution at pH 9. *E*, absorbance spectra of PP-IX (10 μM) in buffers of pH 4.5, 7.4, and 9 supplemented with 1% Empigen BB (Emp), and 100 mM HCl, respectively. *F* and *G*, absorbance spectra of PP-IX (10 μM) in 400 mM phosphate buffer (pH 7.4), supplemented with increasing concentrations of Emp (*F*) or NP-40 (*G*). The traces shown in *panel F* correspond to 0, 0.22, 0.34, 0.38, 0.42, 0.47, 0.52, 0.58, 0.64, 0.98, and 36.76 μM Emp (bottom to top), respectively. *Panel G* shows the absorbance spectra of PP-IX in 400 mM phosphate buffer (pH 7.4) with 0, 0.20, 0.33, 0.51, 0.78, 1.06, 1.31, 1.62, 32.41 μM NP-40 (bottom to top), respectively. Arrows indicate the direction of spectral change on detergent addition. The absorbance spectra shown in *panels A–G* are representative of three independent experiments. *H*, absorbance at 400 nm for the PP-IX solutions described in *panels F* and *G* was plotted as a function of detergent concentration, and the data was fitted to a sigmoidal dose–response curve using GraphPad Prism 8. The plots represent an average of three independent experiments ± standard deviation. *I*, a schematic summary of the findings in Figure 1 that highlight the reversible conversion of PP-IX into monomer, dimer, and higher-order structures as a function of pH and detergent.

PP-IX speciation results in distinct UV–visible and fluorescence emission signatures (3, 4, 5, 43). In aqueous solution, pH and ionic strength are the principal modulators of PP-IX speciation (3). We investigated the nature of PP-IX speciation at pH 7.4 (physiological), pH 4.5 (lysosomal), and pH 9, using previously reported spectra (3, 4, 5, 43) for assignments. UV–visible and fluorescence spectra were collected by diluting freshly prepared PP-IX stock solutions in the indicated buffers (Fig. 1, *A* and *B*). In 100 mM HCl, PP-IX exists as monomers, characterized by the sharp Soret band at 409 nm (Fig. 1*A*, Table 1). At pH 4.5, PP-IX monomers form H-aggregates (higher-order structures), characterized by a broad Soret band with shoulders at 356 and 466 nm. At pH 9 and in 100 mM NaOH, PP-IX exists exclusively as dimers, as judged from the characteristic Soret peak at 380 nm. Notably, the absorbance spectrum at pH 7.4 shows features of both pH 4.5 and 9, displaying a broad Soret band with slightly red-shifted shoulders (379 and 469 nm) compared with the pH 4.5 spectrum (Fig. 1*A*, Table 1). This suggests that at pH 7.4, PP-IX consists of a mixture of higher-order aggregates and dimers. In addition to the changes in the Soret band, changes in the Q-bands in the 500–700 nm region were observed. In 100 mM HCl, where the four pyrrole nitrogens are expected to be protonated, two Q-bands are observed (Table 1). The number of Q bands increases to 3 (pH 4.5, 7.4) and 4 (pH 9) as the extent of protonation decreases (Table 1).

**Table 1:** Summary of UV-vis and fluorescence spectral features of different PP-IX nanostructures

	Absorbance peaks (nm)					Fluorescence peaks (nm)	PP-IX speciation
	Soret	Q4	Q3	Q2	Q1		
[NaOH], 100 mM	381	516	554	579	633	630, 687	Dimer
pH 9	384	517	555	587	640	641, 685	
pH 7.4	379–469	-	542	595	648	631, 670	Dimer, higher-order structures
pH 4.5	356, 466	-	539	597	647	662, 720	Higher-order structures
[HCl], 100 mM	409	-	556	597	-	610, 665	Monomer
pH 9+Emp	408	507	541	581	632	638, 702	
pH 7.4+Emp	408	508	541	580	633	638, 702	
pH 4.5+Emp	408	505	543	579	635	639, 703	
BSA+PP-IX, pH 4.5	355, 469	-	539	597	649	638, 684	BSA+PP-IX higher-order structures
BSA+PP-IX, pH 7.4	391	516	553	585	629	642, 683	BSA+PP-IX dimer
BSA+PP-IX, pH 9	391	509	553	576	627	642, 682	BSA+PP-IX dimer
BSA+PP-IX, Emp	406	504	539	585	629	636, 700	Monomer

The table shows the absorbance and fluorescence peaks from the plots shown in Figure 1, *A*, *B*, and *E*, and Figure 4, *A*–*D*.

Fluorescence emission spectra of the different PP-IX species (Fig. 1*B*) revealed that significant fluorescence quenching is associated with higher-order aggregates of PP-IX. Thus, at pH 4.5, PP-IX displays a minimal fluorescence signal compared with pH 7.4 and 9 (Fig. 1*B*).

We also observed that pH-induced PP-IX speciation is reversible (Fig. 1, *C* and *D*). Thus, the higher-order PP-IX stru ctures observed at pH 4.5 converted to a mixture of higher-order structures and dimers (pH 7.4) and then dimers (pH 9) as the pH is increased, albeit conversion is incomplete (Fig. 1*C*, see shoulder indicated by arrow). The transition in the reverse direction (dimers → higher-order structures) was observed when the pH 9 PP-IX solution at was progressively acidified to pH 7.4, then 4.5 (Fig. 1*D*).

Earlier we had reported that PP-IX induces protein aggregation in buffers containing detergents (*e.g.*, Empigen BB, NP-40) (36, 37, 39, 42). Given the profound effect of pH on PP-IX speciation, we tested whether detergent plays a role in porphyrin speciation. Notably, irrespective of pH, 1% Empigen BB, a nondenaturing zwitterionic detergent, shifts the equilibrium completely to monomeric PP-IX mimicking the effect of 100 mM HCl (Fig. 1*E*). To probe the underlying mechanism, PP-IX (10 μM) in pH 7.4 phosphate buffer was supplemented with increasing concentrations of Empigen BB (Fig. 1*F*) or NP-40, a nonionic nondenaturing detergent, followed by recording of the absorbance spectra (Fig. 1*G*). Both detergents lead to transition of PP-IX higher-order structures and dimers to monomers (Fig. 1, *F* and *G*). The absorbance at 400 nm showed a sigmoidal dependence on detergent concentration, which is characteristic of micelle formation (Fig. 1*H*). Thus, detergent-induced PP-IX monomerization appears to be caused by incorporation of monomers into detergent micelles. In summary, we observed that PP-IX reversibly transitions from dimers (at high pH) to a mixture of dimers/higher-order structures (at neutral pH) to higher-order structures (at low pH), while detergents and pH values of <3 favor PP-IX monomer formation (Fig. 1*I*).

### Smaller PP-IX nanostructures have higher oxidizing potential

**Figure 2:**
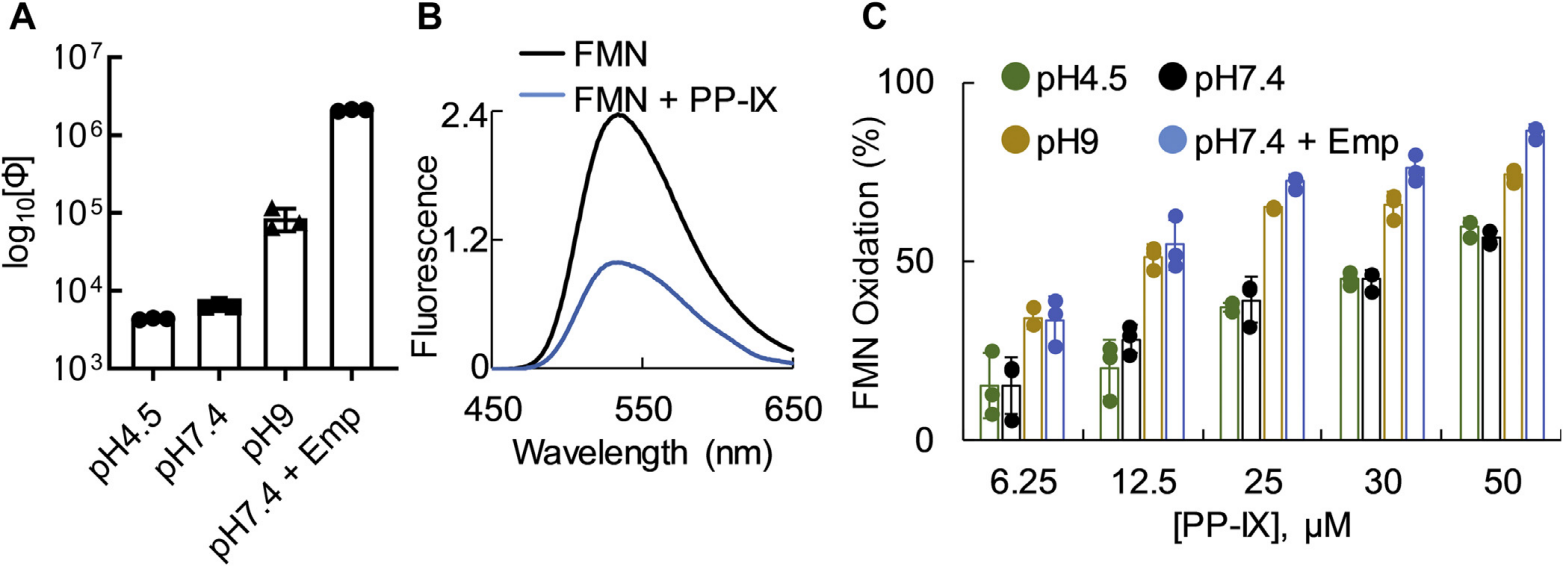
**Smaller PP-IX nanostructures have higher oxidizing potential.***A*, quantum yield of different-sized PP-IX nanostructures (average of three independent experiments ±standard deviation). All five combinations showed statistically significant (*p* < 0.05) difference, except pH 4.5 *versus* pH 7.4 (see Table S1). *B*, fluorescence emission spectra of FMN (21 μM) ± PP-IX (50 μM) at pH 7.4 were recorded after exciting the solutions at 400 nm. The data is representative of three independent experiments. *C*, PP-IX-mediated FMN oxidation at the indicated pH and PP-IX concentrations (average of three independent experiments ±standard deviation).

Since higher-order PP-IX structures show profound fluorescence quenching (Fig. 1*B*), we characterized the effect of PP-IX speciation on the quantum yield (Φ) of PP-IX. As the nanostructure size decreases with increasing pH or with detergent, there is a significant increase in quantum yield (Φ) (Fig. 2*A*, Table S1). PP-IX monomers (observed in 1% Emp, pH 7.4) exhibited the highest Φ.

Next, we tested whether the size-dependent increase in Φ translated to an increase in PP-IX-mediated photooxidation. For this assay, we examined PP-IX-mediated oxidative destruction using the chromophore FMN (Fig. 2*B*). PP-IX-mediated FMN oxidation increased as a function of PP-IX concentration (Fig. 2*C*). Importantly, the smaller PP-IX structures (monomers and dimers, observed at pH 9 and pH 7.4 + Emp) caused significantly greater FMN oxidation (Fig. 2*C*, Table S2). In contrast, there was no significant difference in the oxidizing capacity of PP-IX at pH 4.5 and 7.4. These data suggest that there are two groups of PP-IX nanostructures with different oxidizing potentials. One group consists of the higher-order structures that exist at pH 4.5 and 7.4, while the second group consists of monomers/dimers at pH 9 or in detergent-containing buffer (pH 7.4).

### PP-IX nanostructure size modulates the type and level of PP-IX-mediated protein aggregation

**Figure 3:**
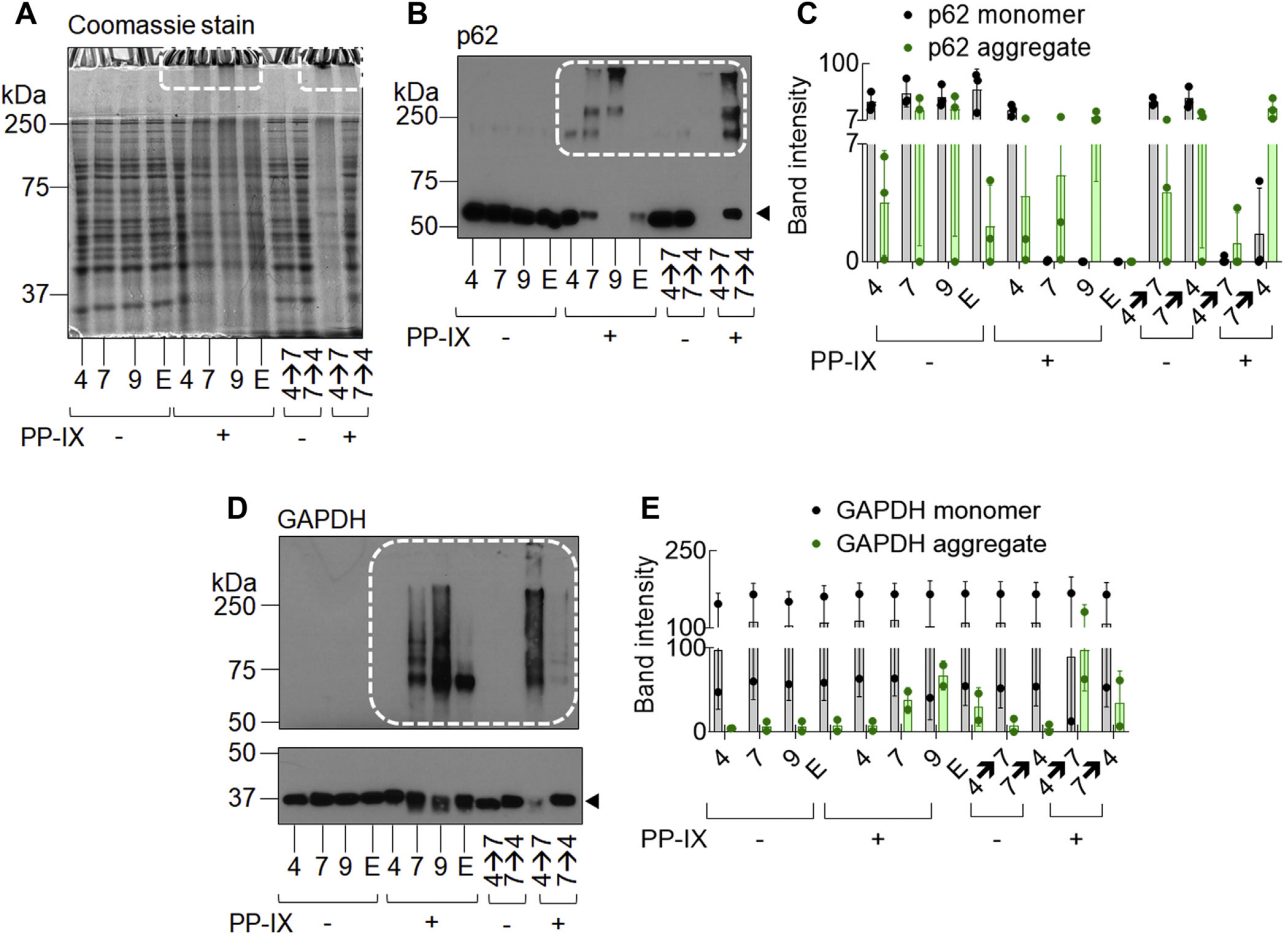
**PP-IX nanostructure size modulates PP-IX-mediated protein aggregation.***A*, the soluble fraction of Huh-7 cell lysate (detailed in Experimental Procedures) was adjusted to 1 mg/ml of protein and treated with 50 μM PP-IX at different pH (“4” = pH 4.5, 7 = pH 7.4, “9” = pH 9, and “E” = pH 7.4 + 1% Empigen). In addition, an aliquot of the pH 4.5 reaction mixture was adjusted to pH 7.4 (“4→7”) and incubated an additional 30 min. Similarly, the pH 7.4 reaction mixture was adjusted to pH 4.5 (7→4). After treatment, the reaction was quenched by adding reducing SDS-PAGE sample buffer. For each lane, 10 μg of protein was separated by SDS-PAGE and stained with Coomassie blue. *B*, reaction mixtures described in *panel A* were separated by SDS-PAGE, transferred to a PVDF membrane, then blotted with anti-p62 antibody. *C*, quantification of monomer (*arrowhead*) and HMW aggregates (*dotted line*) band intensity was done by densitometric scanning of the autoradiograph using ImageJ software, n = 3, mean ± standard deviation. *D*, same as *panel B*, except that the PVDF membrane was blotted with anti-GAPDH antibody. The monomer portion of the blot (*lower panel*, *arrowhead*) was exposed for 5 s while the aggregate (*upper panel*, *dotted line*) was exposed for 15 min using the same PVDF membrane. *E*, same as *panel C*, n = 2, mean ± standard deviation.

Next, we tested the hypothesis that the increased oxidizing potential of PP-IX dimers/monomers leads to increased protein aggregation. PP-IX-mediated high-molecular-weight (HMW) aggregates that failed to enter the SDS-PAGE resolving gel (Fig. 3*A*, *dotted boxes*) were more prominent in the PP-IX-treated samples as the pH was increased from 4.5 to 9.

Two soluble proteins, sequestosome 1 (p62) and glyceraldehyde 3-phosphate dehydrogenase (GAPDH) also exhibited a dramatic increase in aggregation as the pH increases (Fig. 3, *B* and *D*, *dotted boxes*, see Fig. 3, *C* and *E* for quantification) with a corresponding loss of monomers at pH 9 (Fig. 3, *B* and *D*, *arrowhead*, see Fig. 3, *C* and *E* for quantification). PP-IX treatment caused three distinct tiers of HMW p62 aggregates (Fig. 3*B*, *dotted box*). Decreasing the size of PP-IX nanostructures (*via* a change from pH 4.5 → pH 9) led to the formation of HMW aggregates. Using pH 7.4 + 1% Empigen, we did not observe HMW aggregates for p62, although there was an evident loss of the monomer (Fig. 3, *B* and *C*, lane “E”+PP-IX, *arrowhead*). We attribute the extensive loss of p62 monomer to loss of antibody reactivity due to protein oxidation and/or aggregation following PP-IX treatment as described previously for lamin B1 (39), cyclin B1, and cdk4 (42). For GAPDH we did not observe a pronounced loss of monomer (Fig. 3, *D* and *E*, *arrowhead*). In terms of HMW aggregate formation, GAPDH showed the trend: pH 9 > pH 7.4 > 1% Emp >> pH 4.5 (Fig. 3*D*, *dotted box*, see Fig. 3*E* for quantification). Notably, in contrast to p62, smaller-sized PP-IX nanostructures [*e.g.*, dimers (pH 9) and monomers (1% Empigen)] lead to formation of smaller-sized HMW structures (Fig. 3*D*, *dotted box*). In summary, these findings highlight the qualitative and quantitative heterogeneity in protein aggregation depending on the speciation of the PP-IX nanostructures and the protein.

### Reversing PP-IX speciation reverses protein aggregation

Since PP-IX nanostructures are interconvertible (Fig. 1, *C* and *D*), we hypothesized that PP-IX-mediated protein aggregation can be reversed by changing the associated PP-IX nanostructure. To test the hypothesis, we titrated PP-IX in pH 4.5 and 7.4 reaction mixtures to pH 7.4 and 4.5, respectively (Fig. 3, *B* and *D*, lanes 4→7 and 7→4, see Fig. 3, *C* and *E* for quantification). Notably, reversing the pH from pH 7.4 to 4.5 increased the population of unaggregated p62 and GAPDH (Fig. 3, *B*–*E*, compare lanes 4→7 and 7→4), which we interpret as disaggregation due to loss of porphyrin binding. The opposite effect was observed upon changing the pH of the incubation mixture from 4.5 to 7.4.

### Serum albumin disrupts PP-IX nanostructures by preferentially binding to PP-IX dimers

**Figure 4:**
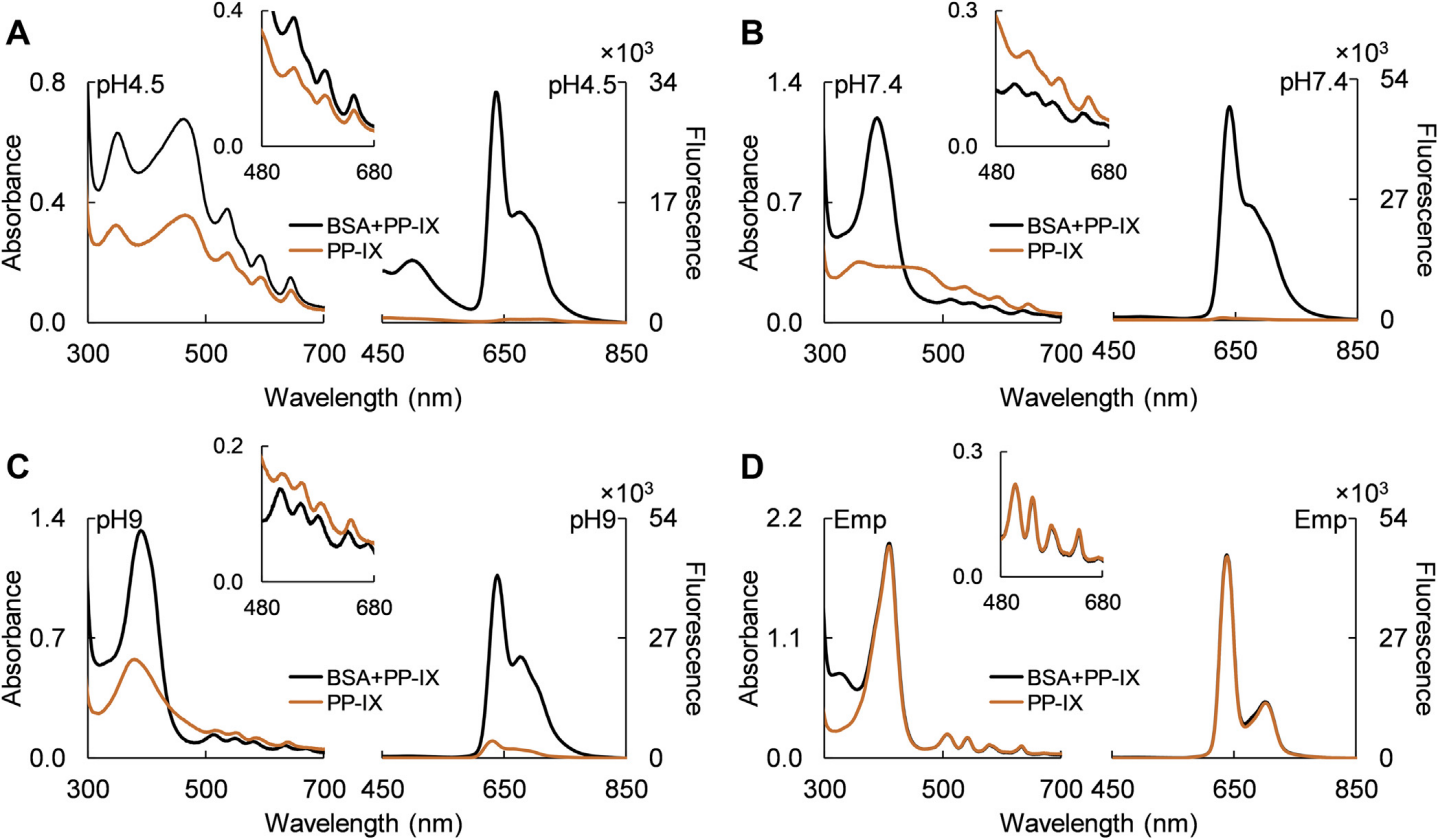
**BSA breaks down higher-order PP-IX nanostructures by preferentially binding to PP-IX dimers.** PP-IX (25 μM) was incubated with BSA (250 μM) at pH 4.5 (*A*), pH 7.4 (*B*), pH 9 (*C*), and with 1% Empigen (Emp) at pH 7.4 (*D*). After a 30 min incubation, the absorbance and fluorescence (excitation 400 nm) spectra were collected. *Insets* show a zoomed portion of the absorbance spectra (480–680 nm) to highlight the changes in the Q-band region. The data is representative of three independent experiments.

We tested how PP-IX speciation modulates its binding to fatty-acid-free bovine serum albumin (BSA), the most abundant serum protein that has also been reported to bind PP-IX (44, 45). PP-IX binding to BSA at pH 4.5, 7.4, and 9 increased the absorbance and fluorescence emission intensity of PP-IX (Fig. 4, *A*–*C*). In contrast, in 1% Empigen (“Emp,” Fig. 4*D*) BSA-PP-IX showed no spectral difference from PPIX, indicating that the PP-IX monomer does not bind to BSA. At pH 4.5, PP-IX binding to BSA increased both the absorbance and fluorescence intensity but did not change the absorbance maxima or peak shape (Fig. 4*A*, Table1). At pH 7.4, a dramatic shift and sharpening of the Soret peak were observed in addition to increased intensity (Fig. 4*B*, Table1), indicating preferential binding of BSA to PP-IX dimers. The increased resolution of the Q-bands from 3 to 4 in the presence of BSA (Fig. 4*B*, main panel and inset, Table1) suggested increased pyrrole ring nitrogen deprotonation and/or decreased mobility due to PP-IX binding to BSA. At pH 9, an increase in intensity was accompanied by a slight red shift in the Soret peak but no changes in the Q-bands (Fig. 4*C*, Table1). The absorbance spectra of the BSA-PP-IX complex at pH 7.4 *versus* 9 were indistinguishable (Table 1 and Fig. S1), indicating that the same dimeric PP-IX was bound. Taken together, our findings suggest that: (i) there are two classes of PP-IX binding sites on BSA, one for PP-IX higher-order nanostructures and a second for PP-IX dimers; (ii) BSA binds preferentially to PP-IX dimers at physiologic pH; and (iii) BSA does not bind to PP-IX monomers.

### PP-IX binding to BSA enhances its oxidizing ability

**Figure 5:**
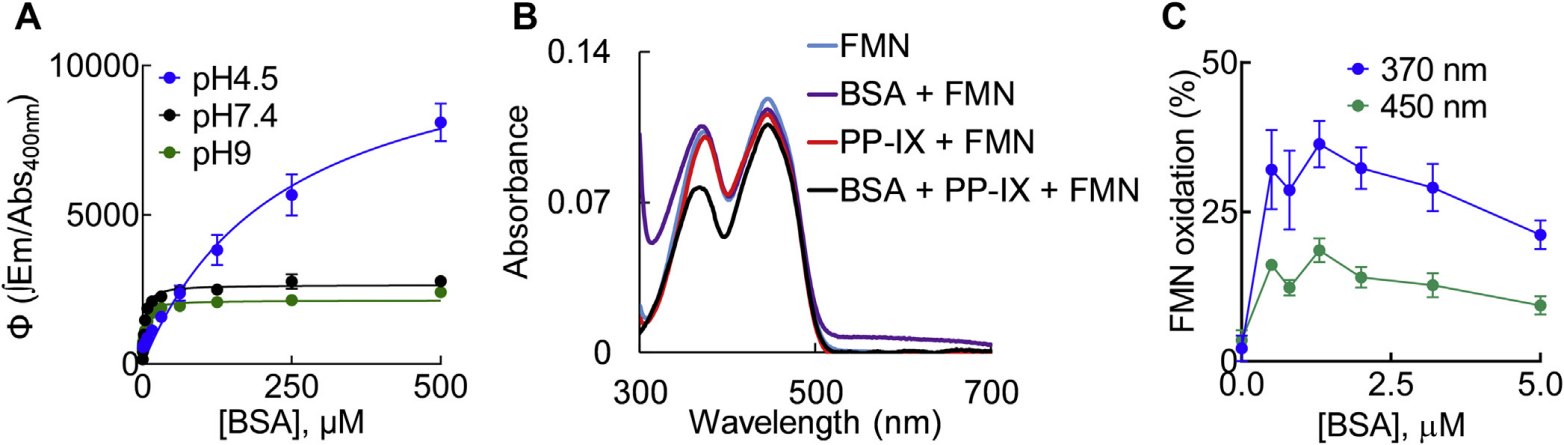
**Protoporphyrin-IX binding to BSA enhances the oxidizing ability of PP-IX.***A*, PP-IX (25 μM) was incubated with increasing concentrations of BSA at the indicated pH, and the quantum yield (Φ) of PP-IX was plotted as a function of BSA concentration. The data was fitted to a hyperbola (*smooth line*) using GraphPad Prism 8. *B*, absorbance spectra of FMN (22 μM), BSA (50 μM) + FMN (22 μM), PP-IX (5 μM) + FMN, and BSA (5 μM) + PP-IX (5 μM) + FMN (22 μM). Displayed data is representative of three independent experiments. *C*, percent change in the absorbance at 370 and 450 nm of the different BSA + FMN + PP-IX mixtures with respect to FMN was plotted as a function of BSA concentration. *Panels A* and *C* show the average of three independent experiments ± standard deviation.

The increase in PPIX absorbance and fluorescence emission upon binding to BSA (Fig. 4) suggests a change in PP-IX Φ upon its binding with BSA. Indeed, when PP-IX was titrated with increasing concentrations of BSA, we observed a concentration-dependent increase in the PP-IX Φ (Fig. 5*A*). We then used FMN oxidation as a readout of whether the oxidizing capacity of PP-IX is enhanced upon binding to BSA. Incubating PP-IX (5 μM) with 0–5 μM of BSA led to a concentration-dependent increase in PP-IX absorbance (Fig. S2) and increased FMN oxidation in the PP-IX+BSA mixture compared with PPI-IX alone (Fig. 5*B*), and this was observed at all BSA concentrations (Fig. 5*C*). At higher BSA concentrations, there was a slight decrease in FMN oxidation, suggesting the ability of BSA to act as a sink for PP-IX-generated oxidants.

### Differential binding affinity of BSA to different PP-IX nanostructures

**Figure 6:**
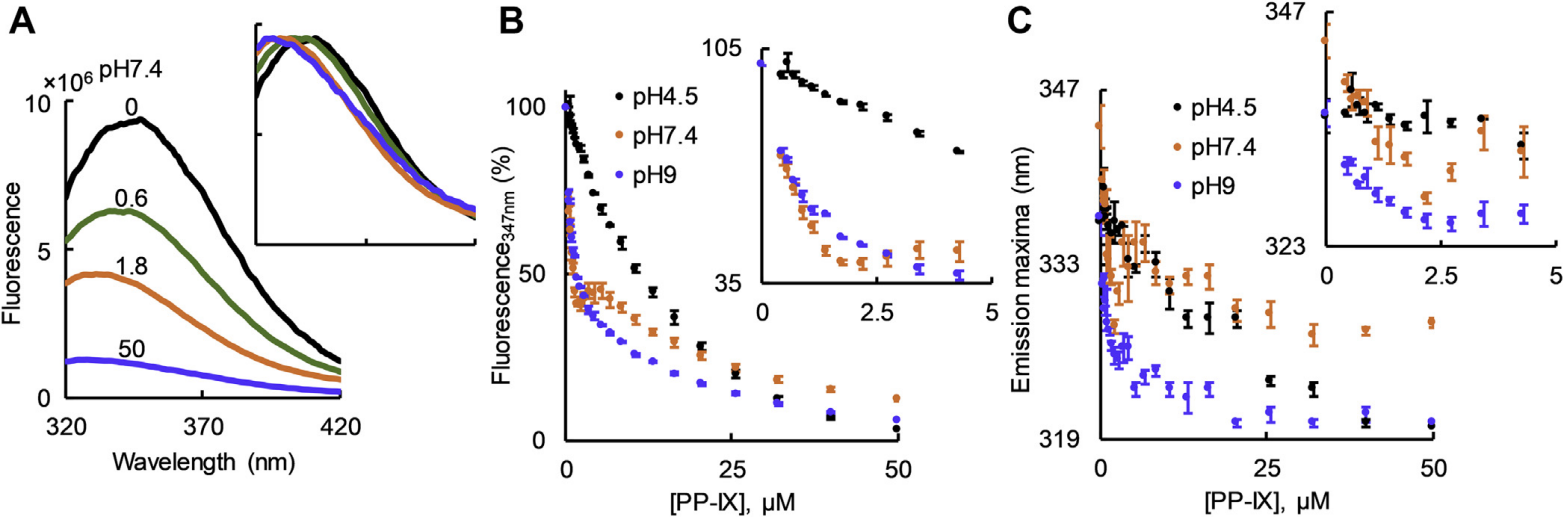
**Protoporphyrin-IX binding to BSA leads to a blue shift and loss of BSA intrinsic fluorescence.***A*, BSA (0.5 μM) was incubated with PP-IX (0–50 μM) for 30 min (pH 7.4), followed by recording of the emission spectra after exciting at 280 nm. The numbers above the spectral traces represent PP-IX concentration in μM. Inset shows the same spectra normalized to the BSA-alone sample emission maxima. *B* and *C*, change in BSA fluorescence emission at 347 nm (*panel B*), and change in emission maxima of BSA (*panel C*) as a function of PP-IX concentration at the indicated pH. For *panel B*, the fluorescence emission of “BSA-alone” was normalized to 100%. *Inset* shows the zoomed initial part (0–5 μM PP-IX) of the curve. The data in *all panels* is an average of three independent experiments ± standard deviation.

**Figure 7:**
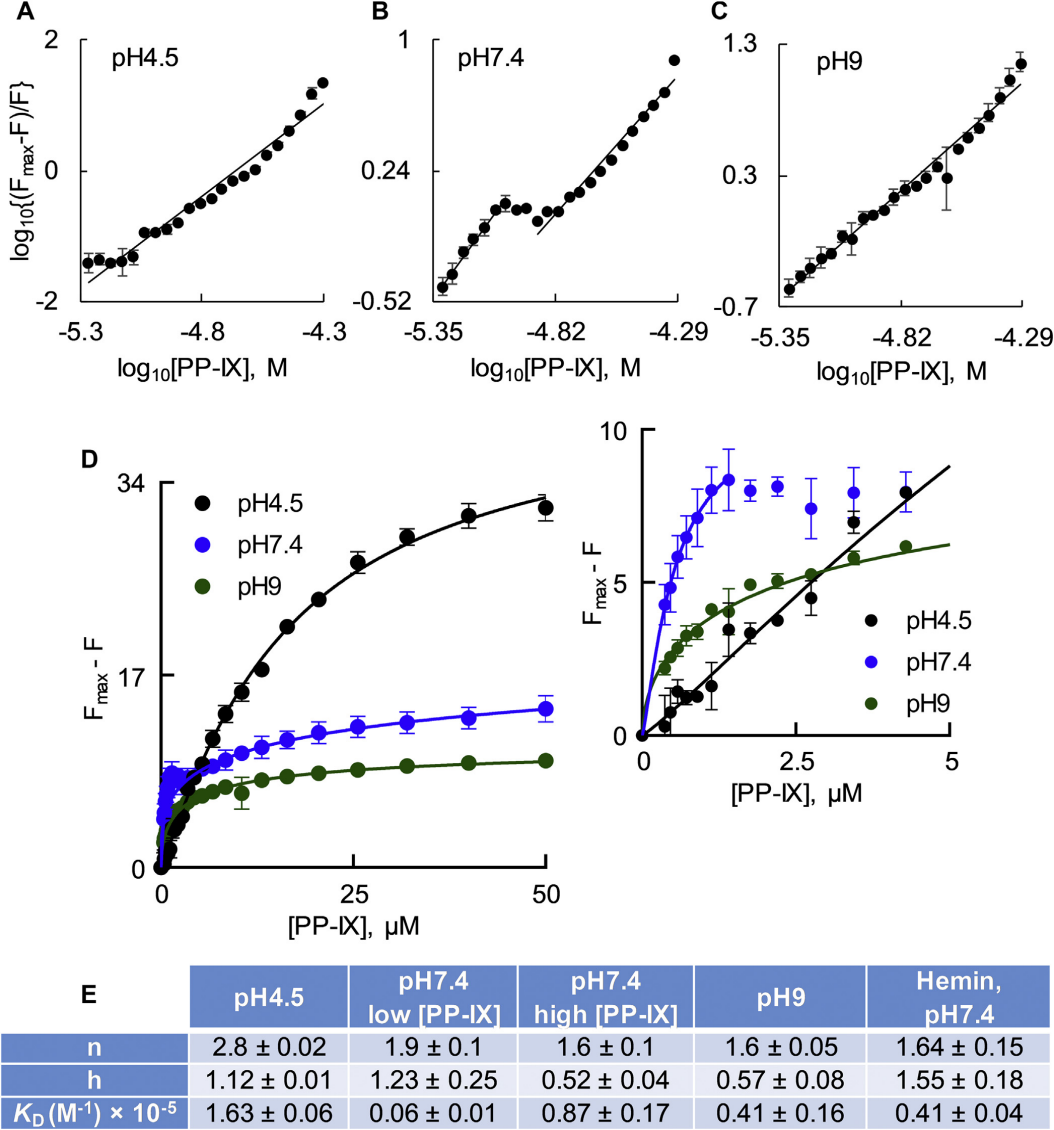
**Fluorescence quenching analyses reveal multiple PP-IX-binding sites on BSA.** Increasing PP-IX concentrations (0–50 μM) were incubated with BSA (0.5 μM) for 30 min (pH 4.5, 7.4, 9). After incubation, quenching of the intrinsic BSA fluorescence was analyzed by measuring BSA florescence emission at 347 nm after exciting at 280 nm. *A–C*, double log plot of log_10_[(F_max_-F)/F] *versus* log_10_[PP-IX], at the indicated pH. *D*, plot of BSA’s F_max_-F as a function of PP-IX concentration. The *smooth lines* show the fitting of the data to a nonlinear regression model of saturation binding—“specific binding with Hill Slope” equation in GraphPad Prism 8. The *inset* of *panel D* shows a zoomed portion of the initial part of the curve in order to highlight the binding profile from 0 to 5 μM PP-IX. The data is the average of three independent experiments ± standard deviation. *E*, binding parameters for BSA+PP-IX and BSA+hemin complexes. n, number of binding sites; h, Hill slope; *K*_D_, dissociation constant. Data shown are average of three independent experiments ± standard deviation.

We used BSA intrinsic fluorescence to monitor conformational changes induced by PP-IX binding and estimated the dissociation constant (*K*_D_) for different PP-IX nanostructures. Although the secondary structure of albumin is reported to not be appreciably different between pH 4 and 9 (46), we examined the intrinsic fluorescence of BSA at pH 4.5, 7.4, and 9 (Fig. S3). The intensity of the fluorescence emission was sensitive to pH, with a small red shift at pH 7.4 (Fig. S3, inset). When BSA was incubated with increasing concentration of PP-IX, two types of changes were observed: (a) concentration-dependent decrease in the emission intensity (Fig. 6, *A* and *B*; Fig.S4), (b) concentration-dependent blue shift (Fig. 6*A*, *inset*, Figs. 6*C* and S4, A and B, *inset*). We attribute the blue shift to the increased hydrophobicity in the environment upon PP-IX binding, with possible oxidation of aromatic residues leading to loss of fluorescence emission. At pH 7.4 (where a mixture of dimers and higher-order oligomers exists), the change in fluorescence intensity at 347 nm and the change in emission maximum showed a biphasic dependence on PP-IX concentration (Fig. 6, *B* and *C*, *insets*). A double log plot of BSA fluorescence quenching by PP-IX also showed a clear biphasic profile at pH 7.4 (Fig. 7*B*). The slope of the steeper part of the curve at pH 7.4 matched that at pH 9, while the second phase matched that at pH 4.5 (Fig .7, *A*–*C*). This suggests that at low PP-IX concentration, PP-IX dimers bind to the high-affinity dimer-binding site on BSA, while at higher PP-IX concentrations, the low-affinity “higher order structure” binding site is occupied. The number of binding sites (n) was calculated from the slopes of the double log plot, and the *K*_D_ was determined by plotting F_max_-F *versus* PP-IX concentration (Fig. 7, *D* and *E*).

To determine whether PP-IX-mediated BSA fluorescence quenching was related to protein oxidation, we examined BSA fluorescence when complexed with hemin (nonphotosensitive porphyrin) and the oxidant hydrogen peroxide (H_2_O_2_). At pH 7.4, hemin binding caused a blue shift and monophasic quenching of BSA fluorescence, similar to PP-IX dimer binding (Fig. S5, *A–C*, Table 1). BSA oxidation by H_2_O_2_ caused a red shift and also led to fluorescence quenching (Fig. S5, *F–H*). LC-MS/MS analysis of the BSA-PP-IX complex at different pH (Tables S3 and S4, Fig. S6) showed consistent oxidation only at H91 with 4–20% of BSA H91 residues being oxidized at pH 9 (Fig. S6*C*). Oxidation of His to Asp/Asn occurs likely *via* a mechanism that involves endoperoxide intermediate formation and subsequent ring opening as previously demonstrated upon photo-oxidation of other proteins (47, 48, 49). Of note, there was no significant BSA aggregation upon PP-IX binding even when the gel was overloaded (Fig. S7).

We conclude that there are two high-affinity PP-IX dimer-binding sites and two low-affinity PP-IX higher-order nanostructure-binding sites per BSA molecule. PP-IX binding to BSA causes aromatic amino acid oxidation–independent fluorescence quenching.

### PP-IX dimers predominantly bind to helical regions of BSA

**Figure 8:**
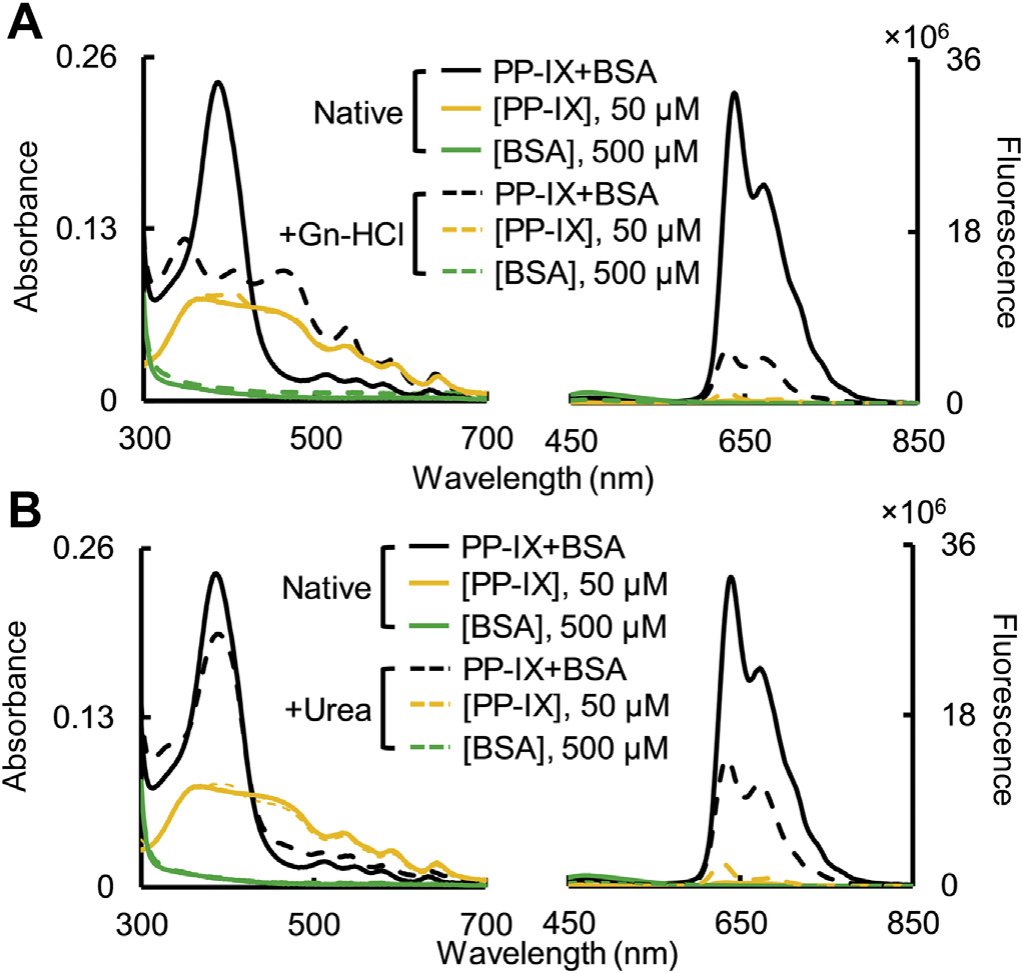
**PP-IX dimers predominantly bind to helical regions of BSA.** BSA (500 μM) was incubated with PP-IX (50 μM) (*solid lines*, “Native”). After treatment, an aliquot of the “Native” BSA, PP-IX and BSA+PP-IX mixtures were denatured, with guanidine-hydrochloride (Gn-HCl; 6.8 M; *panel A*) or urea (*panel B*; 7.2 M), respectively. Absorbance and fluorescence (excitation at 400 nm) spectra were collected for all the samples at the same time. For clarity, the data is shown in two different panels, overlayed with the “Native” samples for comparison. The data is representative of three independent experiments.

To identify which secondary structural element binds PP-IX, we tested the dissociation of BSA-PP-IX complex by urea or guanidine hydrochloride. Guanidine hydrochloride denatures α-helices primarily, while urea affects β-sheets (50). The BSA-PP-IX complex dissociated in the presence of guanidine hydrochloride (Fig. 8*A*), but not in the presence of urea (Fig. 8*B*). Since albumin is predominantly (64%) α-helical (51), and guanidine-HCl was more efficient at dissociating the BSA-PP-IX complex, we conclude that PP-IX dimers preferentially bind to helical parts of the protein. Interestingly, H91 is the only BSA residue that is oxidized by PP-IX, which also falls within an α-helical region. As expected, denaturing BSA with either guanidine-HCl or urea BSA prevented PP-IX binding (Fig. S8).

### BSA protects against intracellular porphyrin accumulation and protein aggregation after ALA + DFO treatment

**Figure 9:**
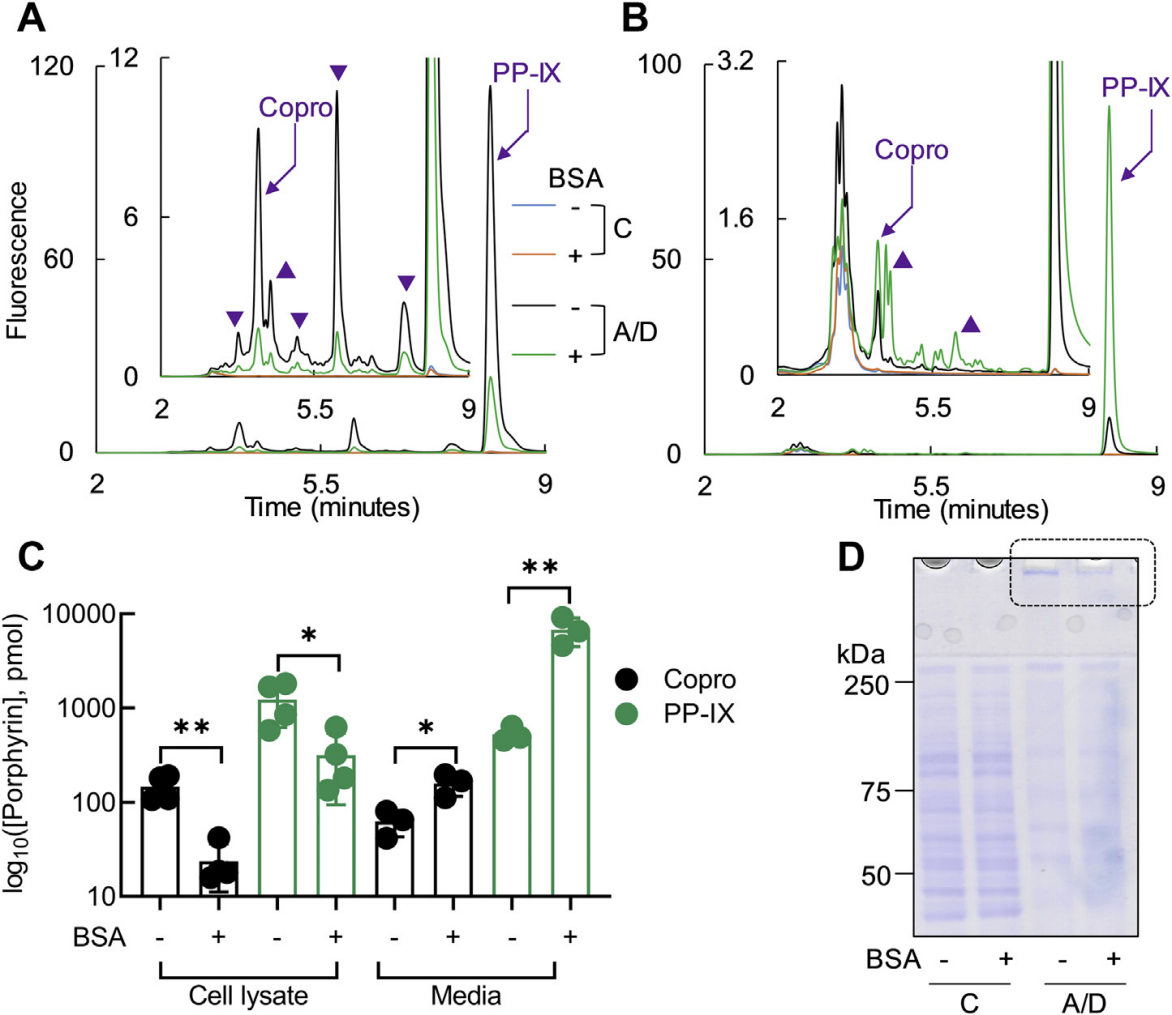
**BSA decreases intracellular porphyrin accumulation after ALA + DFO treatment of HuH-7 cells.** HuH-7 cells were treated with ALA + DFO (A/D) for 16 h in serum-free media (supplemented with 5 mg/ml BSA where indicated). Porphyrins were analyzed in the cells and the media by UPLC. *A* and *B*, UPLC chromatograms show the amounts of fluorescent porphyrins inside the cells (*A*) and in the media (*B*). Copro and PP-IX peaks are labelled, and arrowheads indicate unidentified fluorescent products that are formed after ALA + DFO treatment. *C*, quantification of Copro and PP-IX levels calculated from the area under the curve of the fluorescent peaks shown in *panels A* and *B*. The data are average of three independent experiments with error bars representing the standard error of measurement. Statistical significance was determined using an unpaired *t*-test (two-tailed). ∗*p* < 0.05, ∗∗*p* < 0.01 and denotes comparison with control. *D*, proteins from the ALA + DFO treated cells were separated by reducing SDS-PAGE and visualized by Coomassie staining (*dotted box* highlights the stained aggregates that did not enter the gel). The data is representative of three independent experiments.

Since BSA binds PP-IX, we hypothesized that BSA may act as an extracellular PP-IX trap and thereby modulate PP-IX-mediated intracellular protein aggregation. To test this hypothesis, we treated HuH-7 cells with δ-aminolevulinic acid (ALA)+deferoxamine (DFO) with or without the addition of BSA to the culture medium. ALA+DFO led to intracellular accumulation of PP-IX and Copro and to their extracellular release (Fig. 9, *A*–*C*). In BSA-supplemented media, significantly less porphyrin accumulated inside cells, while more porphyrin was released (Fig. 9, *A*–*C*). We attribute this to the ability of BSA to bind PP-IX and shift the equilibrium of PP-IX secretion.

**Figure 10:**
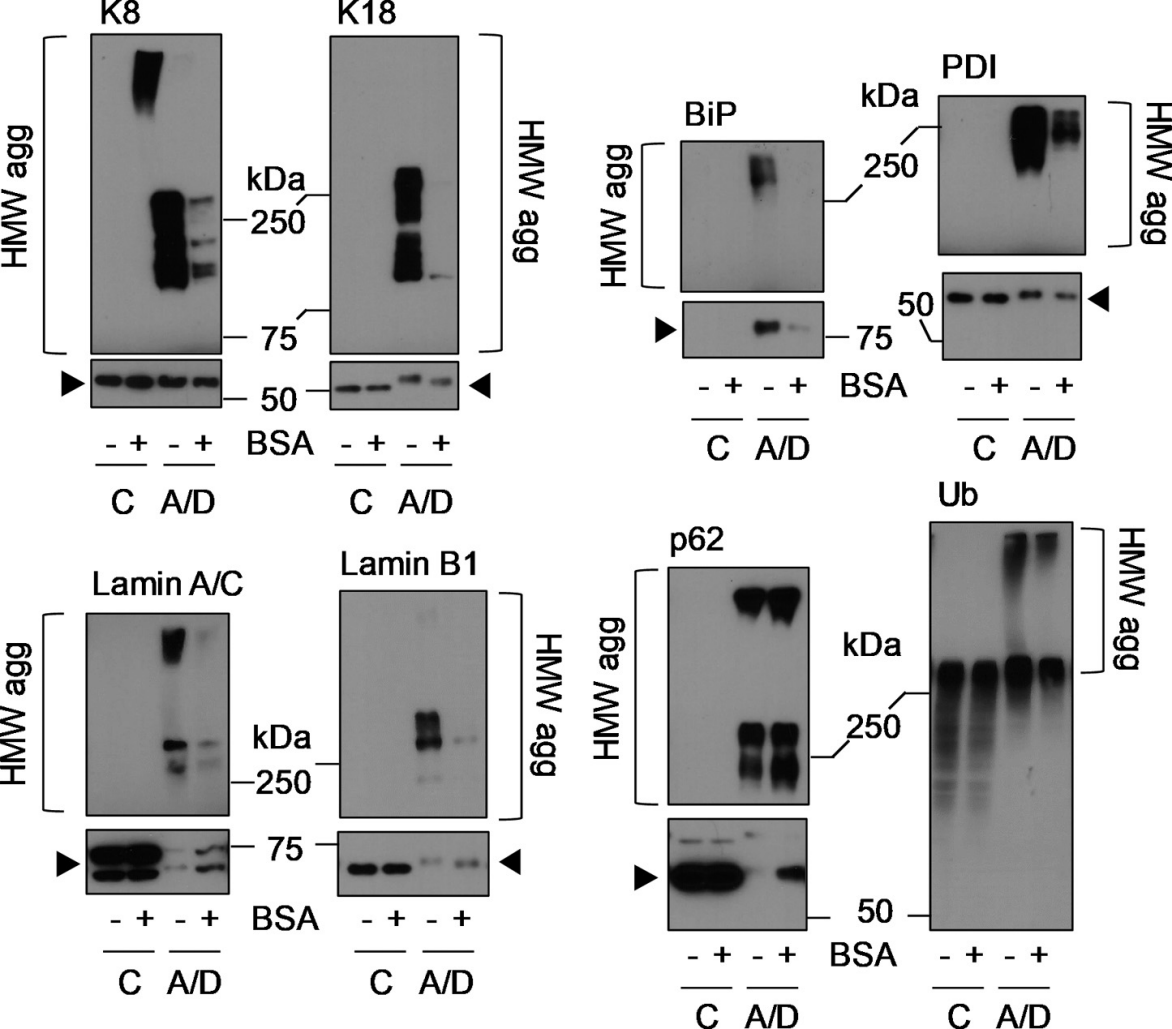
**BSA protects from ALA + DFO-mediated cellular protein aggregation.** Proteins from the experiment described in Figure 9 were separated in reducing SDS-PAGE followed by transferring to PVDF membranes and immunoblotting with antibodies to the indicated antigens. Monomer bands are marked with an *arrowhead* and, where shown separately, the monomer blots (*lower panels*) were exposed for 5 s, while the HMW aggregate blots were exposed for 15 min, from the same PVDF membrane. The data shown is representative of three independent experiments.

ALA+DFO-treated cells with BSA in the medium had lower levels of HMW protein aggregates as seen by Coomassie blue staining (Fig. 9*D*, *dotted box*). We also tested proteins from several subcellular compartments including cytosolic (keratins 8 and 18; K8/K18), endoplasmic reticulum (BiP and PDI), nuclear (lamins A/C and B1), and proteins involved in the degradation and clearance pathways (p62 and ubiquitin). All tested proteins were protected by BSA against ALA+DFO-induced loss of the monomeric form and aggregation (Fig. 10). Among the proteins we tested for aggregation, BiP is upregulated after PP-IX or ALA+DFO treatment (39, 42). BiP is an ER-specific HSP70 isoform and a marker for the unfolded protein response (52). As expected, we observed dramatic BiP upregulation and aggregation after ALA+DFO treatment, both of which were attenuated in the presence of BSA (Fig. 10).

## Discussion

### Targeting PP-IX speciation as a potential therapeutic strategy

Porphyrin-mediated protein aggregation plays a major role in porphyria-associated tissue damage (27, 39, 42). PP-IX oligomerization causes static quenching of the fluorophore (3, 53), leading to dramatically reduced quantum yield and protein aggregation (Figs. 2*A* and 3), highlighting the importance of preventing PP-IX aggregation in order to hinder porphyrin-mediated cell damage. In this context, agents that affect PP-IX speciation could be therapeutically valuable for porphyria. Drugs targeting heme speciation are already known. For example, antimalarial quinolone compounds prevent hemozoin granule formation by binding to heme monomers, and the heme monomer–quinolone complex is incorporated at the elongating hemozoin polymer, thereby inhibiting polymer elongation (54, 55). Similarly, caffeine has been reported to prevent heme aggregation by forming a heme–caffeine monomeric complex (56). Of note, PP-IX and other related porphyrins are currently used as antitumor agents in photodynamic therapy (PDT) whereby porphyrin-mediated protein aggregation and proteotoxicity play a beneficial cytotoxic role. For example, verteporfin, a PDT agent, causes tumor selective protein aggregation (38). Thus, modulation of PP-IX speciation in a context-dependent manner represents an attractive strategy to attenuate or enhance its toxicity.

### Selectivity and specificity of PP-IX-protein interaction and related protein aggregation is guided by PP-IX speciation

A hallmark of PP-IX-induced protein aggregation is its selectivity. Some known PP-IX-binding proteins, such as translocator protein (TSPO) and fatty acid binding protein 1 (FABP1), do not aggregate (39). Here we observed that BSA does not bind PP-IX monomers (Fig. 4). We posit that PP-IX speciation might underlie some of the selectivity of PP-IX-protein interactions and protein aggregation. As we demonstrate (Fig. 3), detergent micellarized-PP-IX monomers lead to qualitatively and quantitatively different protein aggregates as compared with those formed by PP-IX dimers and higher-order structures. Additionally, PP-IX-mediated protein aggregation is organelle-selective. Proteomic analysis of protein aggregation of livers in ALA+DFO-injected zebrafish showed aggregation of cytosolic, nuclear, ER, and mitochondrial proteins but absence of lysosomal protein aggregation (37). Lysosomes accumulate porphyrins similar to the cytosol, mitochondria, ER, and nucleus (57, 58, 59, 60). However, lysosomes are acidic compartments with a luminal pH of 4.5–5 (61), which aligns with our finding that PP-IX exists as higher-order and relatively inert structures with lower oxidizing and protein aggregating ability at the lysosomal pH. Furthermore, porphyrin oligomers have lower quantum yield and impaired ^1^O_2_ generation capacity (62). Indeed, live cell imaging of ^1^O_2_ demonstrated that despite lysosomal and mitochondrial accumulation of porphyrin, ^1^O_2_ was generated only in the mitochondria (63). Thus, accumulation of anionic porphyrins such as PP-IX, in the acidic environment of lysosomes (60), is not accompanied by protein aggregation. In contrast to higher-order PP-IX structures, PP-IX monomerization increases its toxicity as shown for detergent monomerized PP-IX, which has a higher oxidizing potential (Figs. 2 and 3). In the liver, bile salts act as surfactant detergents and, by virtue of their ability to solubilize PP-IX, play an important role in porphyrin clearance. In fact, several mouse models of protoporphyria show the important role of bile salt-PP-IX interaction in the development of cholangiopathy (64, 65).

### Porphyrin-binding proteins as a PP-IX speciation agent

**Figure 11:**
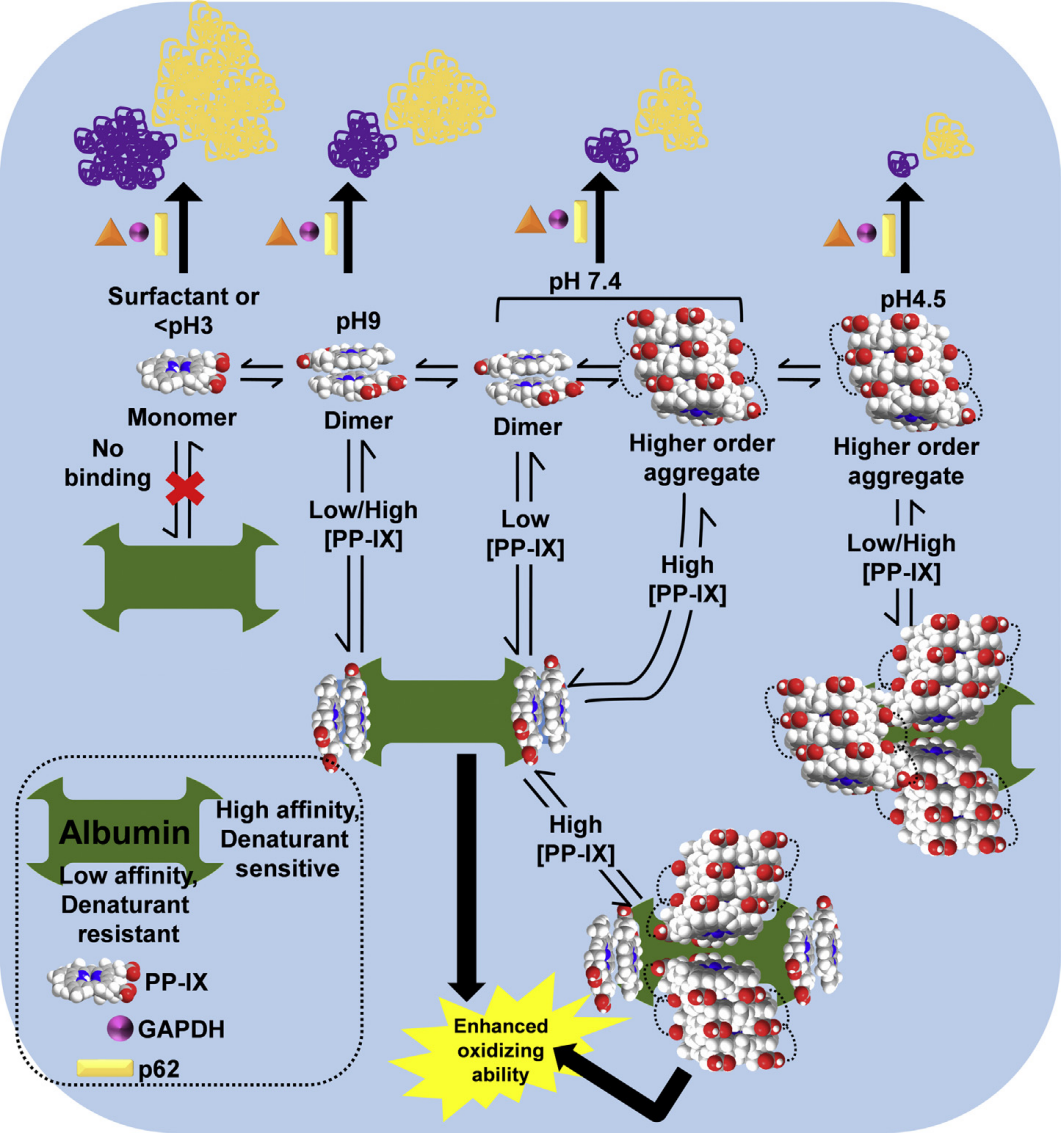
**Schematic that summarizes the effect of PP-IX nanostructures in PP-IX-mediated protein aggregation and modulation of PP-IX speciation by albumin.** PP-IX spontaneously speciates into different-sized nanostructures depending on the pH of the solution. PP-IX monomers and dimers have higher oxidizing and protein aggregating ability than higher-order PP-IX nanostructures. Serum albumin has two classes of PP-IX-binding sites: (a) high-affinity PP-IX dimer-binding site, and (b) low-affinity PP-IX higher-order structure binding site. BSA solubilizes higher-order PP-IX nanostructures to PP-IX dimers, by preferentially binding PP-IX dimers, which subsequently increases PP-IX oxidizing ability.

Binding to albumin enhances PP-IX toxicity and increases its oxidizing ability (Fig. 5) by shifting the equilibrium from higher order to dimeric PP-IX (Fig. 9). On the other hand, albumin acts as a “PP-IX sponge” and decreases endogenous cellular PP-IX accumulation (11, 12, 13) and protein aggregation (Figs. 10 and 11). Our findings raise the question whether albumin modulates PP-IX-induced protein aggregation intracellularly in hepatocytes or extracellularly in serum. Thus, by modulating speciation, PP-IX-binding proteins such as albumin might act as novel genetic modifiers of porphyric tissue damage.

### Impact of the albumin–PP-IX interaction

Albumin constitutes nearly 50% of total plasma protein content (66). It is synthesized in hepatocytes, then secreted into the circulation (66, 67) where it maintains oncotic pressure (68). In addition, albumin performs diverse functions ranging from ligand transport to serving as an antioxidant (66, 67, 68, 69). Our data show that PP-IX binding leads to BSA oxidation at His91 and a conformational change (Figs. 5 and 7; Fig. S7). Since albumin also binds to a vast range of proteins in plasma [the “albuminome” (70, 71, 72)], PP-IX binding could lead to disruption of components in the “albuminome.” In fact, oxidized human serum albumin is reported to differ significantly as compared with its unoxidized counterpart with respect to ligand binding and antioxidant properties (73).

## Conclusion

The reversible nature of PP-IX speciation (Fig. 11) and their different functional properties provide a potential target for therapeutic interventions. Our findings demonstrate that PP-IX-induced protein aggregation could be reversed simply by a pH-induced shift in PP-IX speciation. This implies that transport of PP-IX-protein aggregates to the acidic lumen of autophagolysosome can shift the equilibrium to higher-order structures and lead to disaggregation of the PP-IX-bound proteins. This highlights the dramatic difference in property and behavior of PP-IX, depending on its degree of oligomerization.

Although porphyrin speciation had been widely studied, there are no reports on the effect of porphyrin speciation in porphyria. Indirect support of porphyrin speciation modulating porphyria-associated tissue damage comes from findings involving uroporphyrin (Uro) in a congenital erythropoietic porphyria model (74). Uro, being hydrophilic, does not self-associate into higher-order structures, so in contrast to PP-IX, it binds to hydroxyapatite and accumulates in the extracellular matrix of osteoblasts. This underscores the importance of studying PP-IX speciation and the protein binding properties of such species in porphyrias, and suggests that modulating PP-IX speciation could be useful for designing effective therapeutics for porphyria and improved PDT agents.

**Figure 12:**
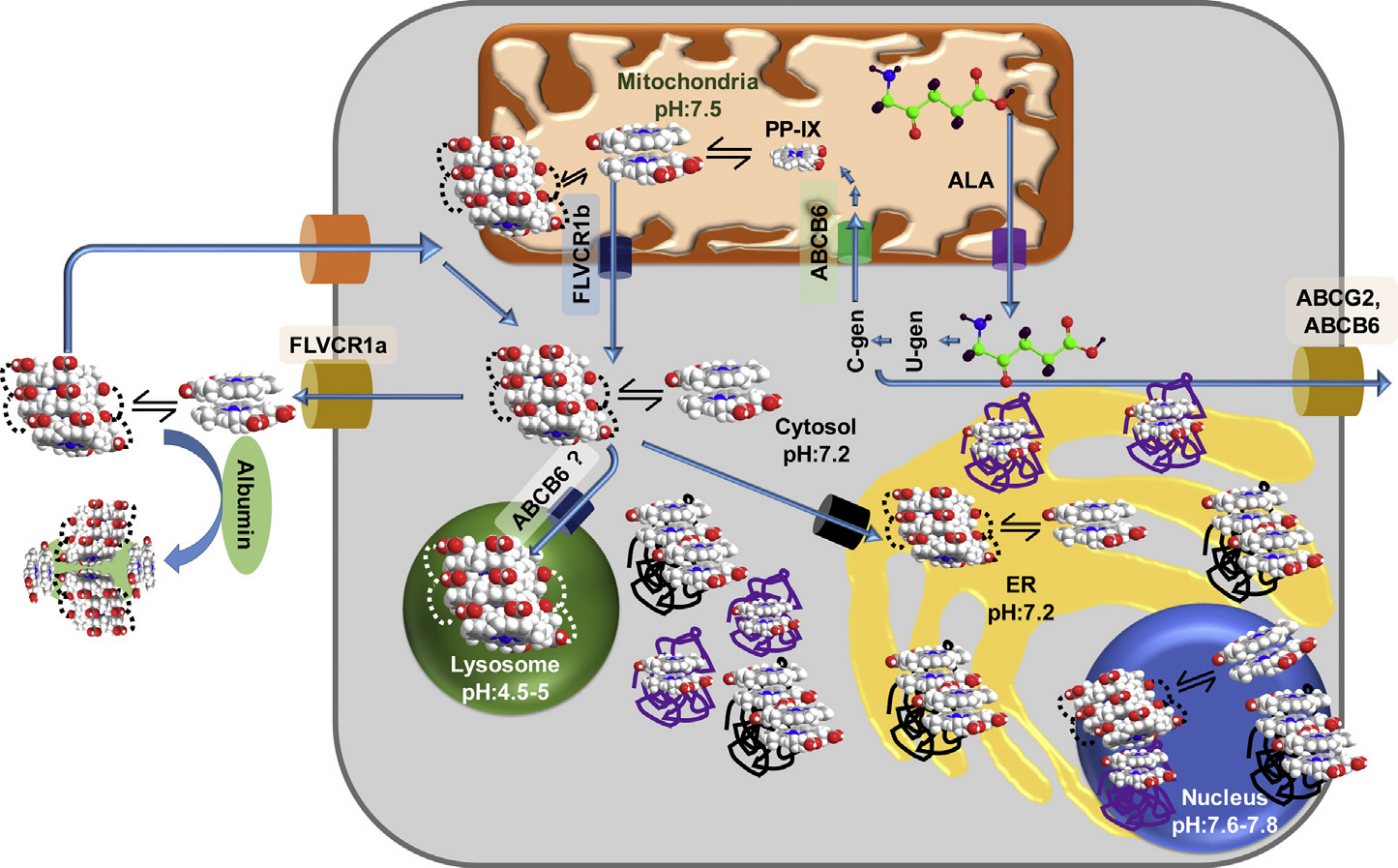
**A model depicting the role of albumin as an extracellular PP-IX trap, thereby modulating PP-IX-mediated proteotoxicity and cellular damage.** PP-IX biosynthesis is initiated in the mitochondria through a multistep mechanism, and is excreted by transporters (FLVCR1a, ABCG2, ABCB6). When the rate of PP-IX synthesis exceeds the rate of PP-IX excretion, PP-IX accumulates intracellularly thereby causing organelle-selective protein aggregation. Lysosomes are protected from PP-IX-mediated protein aggregation since PP-IX speciates into biologically inert higher-order structures in its acidic pH. Extracellular PP-IX-binding proteins, such as with albumin, may serve as a “PP-IX sponge” and shift the equilibrium of PP-IX excretion. Thus, in the presence of albumin, there is less intracellular PP-IX retention and protein aggregation. C-gen, coproporphyrinogen; U-gen, uroporphyrinogen.

The intracellular PP-IX homeostasis is maintained by a concerted action of PP-IX transporters and PP-IX biosynthesis (Fig. 12). Our findings indicate that albumin, by acting as a “PP-IX sponge,” shifts the equilibrium of PP-IX homeostasis. This subsequently decreases intracellular PP-IX retention and ensuing protein aggregation (Fig. 12). In addition, albumin is oxidized at H91 into both Asn and Asp upon binding to PP-IX. This could have applications such as chemical mutagenesis.

## Experimental Procedures

### Cell lines and reagents

The human hepatocellular carcinoma cell line, HuH-7 cells (originally from the Japanese Collection of Research Bioresources Cell Bank), was a kind gift from Dr Lei Yin (University of Michigan). HuH-7 cells were grown in Dulbecco’s modified Eagle’s medium (Cellgro), supplemented with 10% fetal bovine serum (Sigma-Aldrich).

PP-IX, deferoxamine mesylate (DFO), 5-ALA hydrochloride, N,N-dimethylacetamide (DMA), NaH_2_PO_4_, Na_2_HPO_4_, flavin mono nucleotide (FMN), and ALA were obtained from Sigma Aldrich. Fatty-acid-free BSA, which was used in all the BSA-related experiments, was from Calbiochem;coproporphyrin-III dihydrochloride (Copro) and uroporphyrin-III dihydrochloride (Uro) were obtained from Frontier Scientific.

Absorbance/fluorescence measurements: PP-IX absorbance and fluorescence measurements were carried out using a TECAN SAFIRE II microplate reader running XFLUOR4SAFIREII (Version: V 4.62n) or the Spectramax id3 running SoftMax Pro.

Quantum yield calculation: Absorbance (from 300 to 700 nm) and fluorescence emission spectra of 50 μM of PP-IX solution were collected. Relative quantum yield (Φ) was calculated using (Φ=∫Em/Abs), where ∫Em is the area under the curve for the emission spectra, and Abs is the absorbance at the excitation wavelength. ∫Em was calculated by integrating the emission spectrum using GraphPad Prism software (GraphPad Software). The following excitation and emission wavelengths were used: excitation at 437 nm, emission at 460–850 nm (pH 4.5); excitation at 368 nm, emission at 400–850 nm (pH 7.4); excitation at 389 nm, emission at 410–850 nm (pH 9); excitation at 410 nm, emission at 450–850 nm (1% Empigen in pH 7.4).

FMN Oxidation: An FMN stock solution (2.2 mM) was freshly prepared in 5 mM phosphate buffer (PB), pH 7.4, and stored away from light, on ice. The FMN stock solution was diluted to a final concentration of 22 μM in 400 mM of NaH_2_PO_4_ (pH 4.5), PB (pH 7.4), Na_2_HPO_4_ (pH 9.0), or 1% Empigen BB in PB (pH 7.4) and incubated with different concentrations of PP-IX. DMA in all the reaction mixtures was maintained at 2.8% (v/v). The control reaction mixture contained FMN (22 μM) in 2.8% DMA in pH 4.5, 7.4, or 9 buffers. After incubating the reaction mixtures (30 min, ambient light), the fluorescence emission between 500 and 700 nm was recorded using a SAFIRE II plate reader, after exciting at 400 nm. The percent oxidation of FMN was estimated from the loss of FMN fluorescence at 533 nm after PP-IX treatment. Additionally, where indicated, PP-IX-mediated FMN oxidation was assayed as above, but in the presence of the indicated concentrations of fatty-acid-free BSA. The extent of FMN oxidation was estimated by the loss of absorbance at 370 and 450 nm.

Treatment of HuH-7 cell lysates with PP-IX and biochemical analysis: Detergent-free HuH-7 cell lysates were prepared by five cycles of freeze-thawing then using a Dounce homogenizer in 5 mM PB (pH 7.4) containing protease inhibitor cocktail (ThermoScientific). The resulting cell extract was centrifuged (14,000*g*, 10 min, 4 °C), and the supernatant and pellet were separated. Protein content in the supernatant was determined using a bicinchoninic acid (BCA) assay. The final reaction mixture contained: 1 mg/ml protein with or without 50 μM PP-IX in 400 mM of NaH_2_PO_4_ (pH 4.5), PB (pH 7.4), Na_2_HPO_4_ (pH 9.0), or 1% Empigen BB in PB (pH 7.4). Control samples had the same concentration of the vehicle, DMA. After incubating (30 min, 37 °C, ambient light), the reaction was stopped by the addition of Laemmli sample buffer. Proteins (10 μg/condition) were separated by SDS-PAGE and stained with Coomassie blue or transferred to polyvinylidene fluoride (PVDF) membranes for immunoblotting. Immunoblots were visualized using horseradish peroxidase–tagged secondary antibody and chemiluminescence (Clarity Western ECL Substrate; BioRad). The antibodies used included those directed to lamin A/C, ubiquitin (Santa Cruz Biotechnology, Inc); lamin B1, p62 (Abcam); K8 (clone TS1) and K18 (clone DC10), protein disulfide isomerase, and BiP (Cell Signaling Technology).

Calculation of number of PP-IX binding site on BSA and BSA-PP-IX dissociation constant: Fatty-acid-free BSA (0.5 μM) was incubated with increasing concentrations of PP-IX (0–50 μM), at pH 4.5, 7.4, and 9 buffers, respectively (30 min). Then, the solution was excited (280 nm) and the fluorescence emission spectra were collected from 320 to 420 nm.

Data were analyzed using a double log plot of ${\log\;}_{10}\left[\frac{\mathrm{Fmax}-F}{\mathrm{Fmax}}\right]$ *versus* ${\log\;}_{10}\left[\mathrm{PP}-\mathrm{IX}\right]$ , where F_max_ and F are the fluorescence of BSA (347 nm) in the absence and presence of PP-IX (75). The plot yielded a straight line, and the number of binding sites was calculated using:

$${\log\;}_{10}\left[\frac{\mathrm{Fmax}-F}{\mathrm{Fmax}}\right]={\log\;}_{10}K+n{\log\;}_{10}\left[\mathrm{PP}-\mathrm{IX}\right]$$

where *K* is the binding constant, and *n* is the number of binding sites.

The dissociation constant (K_D_) and Hill coefficient (*h*) were calculated by plotting F_max_-F *versus* [PP-IX] and fitting the data to “Specific binding with Hill slope” equation in GraphPad Prism. For n, K_D_, and h calculations, traces from each experiment were fitted individually and then averaged.

Mass spectrometric analysis of PP-IX-mediated BSA oxidation: BSA (10 μM) was treated with 500 μM PP-IX in pH 4.5, 7.4, and 9 buffers (30 min, 22 °C). Then, 100 μl of the reaction mixture was extracted by mixing with 850 μl of methanol and 50 μl of 0.1 N perchloric acid. The resulting suspension was pelleted (20,800*g*, 4 °C, 20 min). The pellet was solubilized in 8 M guanidine hydrochloride, 50 mM Tris HCl (pH 8.1), 20 mM DTT and incubated (60 °C, 30 min), followed by incubation (in the dark, 22 °C) with 40 mM of iodoacetamide to block the reduced cysteines. A fraction of the sample (10%) was desalted using ziptip C4 (EMD Millipore) and dried under vacuum. The sample was treated with 0.2 μg trypsin in 10 μl of 50 mM NH_4_HCO_3_ and incubated overnight at 37 °C. The digested peptides were acidified with 1 μl of 10% formic acid followed by analysis using liquid chromatography–tandem mass spectrometry (LC-MS/MS). Samples were analyzed with a Q Exactive HF tandem mass spectrometer coupled to a Dionex Ultimate 3000 RLSCnano System (Thermo Scientific) by loading onto a fused silica trap column Acclaim PepMap 100, 75 μm × 2 cm (ThermoFisher). After washing (5 min, 5 μl/min with 0.1% TFA), the trap column was brought in-line with an analytical column (Nanoease MZ peptide BEH C18, 130 Å, 1.7 μm, 75 μm × 250 mm, Waters) for LC-MS/MS analysis. Peptides were eluted using a segmented linear gradient from 4 to 90% B (A: 0.2% formic acid, B: 0.08% formic acid, 80% acetonitrile): 4–15% B (5 min), 15–50% B (50 min), and 50–90% B (15 min). Mass spectrometry data was acquired using a cyclic series of a full scan from 250 to 2000 with resolution of 120,000 followed by MS/MS (HCD, relative collision energy 27%) of the 20 most intense ions (charge +1 to +6) and a dynamic exclusion duration of 20 s. For database search, the peak list of the LC-MS/MS was generated by Thermo Proteome Discoverer (v. 2.1) into MASCOT Generic Format (MGF) and searched against the Uniprot bovine database plus a database composed of common laboratory contaminants using an in-house version of X!Tandem (GPM Fury) (76). The number of entries in the database searched included: # of bovine sequences 32,319 and # of contaminant sequences 245. The release version of sequence database was the last update of bovine albumin sequence (July 03, 2018). The search parameters were as follows: fragment mass error (20 ppm), parent mass error (±7 ppm), fixed modification:C-carbamidolethyl; flexible modifications: Met oxidation for the primary search and other modifications were done at the refinement stage and less than three modifications are set for each search. Protease specificity: trypsin (miss two cuts) and only spectra with log_e_ < −2 were included in the final report. A detailed list of the oxidative modifications is given in Table S4. Initial filtering of the data was performed by using Skyline software to detect all peptides with modifications listed in Table S4. Peptides that were preliminarily identified by the Skyline software were validated by searching for their MS/MS spectra. Only modifications that were reproducibly observed in all three experimental trials were considered.

Treatment of HuH-7 cells with ALA+DFO ± BSA: HuH-7 cells were seeded in 10 cm dishes in complete (serum containing) media. Upon cell confluency, the media was replaced with serum-free media with ALA+DFO (1 mM/100 μM) ± fatty-acid-free BSA (5 mg/ml). Control dishes had serum-free media ± fatty-acid-free BSA (5 mg/ml). After 16 h, the entire volume of medium was collected and then pelleted (3000*g*, 10 min) to remove floating cells and debris. The supernatant was saved for analysis. For the remaining adherent cells, the culture plates were rinsed twice with PBS, and the cells were harvested by scraping in 10 ml PBS. The resulting cell suspension was centrifuged (400*g*, 5 min) followed by lysis of the cell pellet with PBS supplemented with 2% SDS, then analysis by SDS-PAGE. The cell lysate and culture medium were also analyzed for porphyrin content as described below.

Ultra performance liquid chromatography (UPLC) analysis of porphyrins: A Waters ACQUITY UPLC system equipped with Empower software, ACQUITY H-Class PLUS (CH-A) Core, comprising of Quaternary Solvent Manager (QSM), a Sample Manager with Flow-Through Needle (SM-FTN), ACQUITY UPLC PDA Detector, ACQUITY UPLC Fluorescence Detector. A reverse-phase octadecylsilica (C18) ACQUITY UPLC BEH Shield RP18 Column, 130 Å, 1.7 μm, 3 mm X 100 mm UPLC column and ACQUITY UPLC BEH Shield RP18 VanGuard Pre-column, 130 Å, 1.7 μm, 2.1 mm X 5 mm was used. The fluorescence detector was set for excitation at 400 nm and recording emission at 635 nm. The column was eluted at a flow rate of 0.5 ml/min with linear gradients of solvents A and B (A, 0.1% formic acid in water; B, 0.1% formic acid in methanol). The solvent gradient was as follows: 0–1 min, 50–50% B; 1–2 min, 50–85% B; 2–3 min, 85–90% B; 3–9 min, 90–95% B; 9–9.5 min, 95–50% B; 9.5–14.5 min, 50–50%. The cells were lysed after ALA + DFO treatment in PBS supplemented with 2% SDS. To extract porphyrins, DMA and 0.1% formic acid in methanol were added to the cell lysate or the isolated culture medium in a 1:1:2 ratio. After mixing, then centrifugation (20,800*g*, 5 min) to precipitate the proteins, 50 μl of the supernatant was injected into the UPLC system. Retention times of PP-IX, Uro, and Copro were determined using commercially obtained standards.

### Porphyrin solution preparation

PP-IX and hemin were dissolved in N,N-dimethylacetamide at 1.77 mM stock solutions, while Uro and Copro were dissolved in 100 mM NaOH, to prepare 5 mM stock solutions. Care was taken not to expose the stock solutions to light. The stock solutions were diluted in appropriate buffers immediately prior to use. ALA and DFO were dissolved in water to yield 500 mM and 50 mM stock solutions, respectively, then filter-sterilized, aliquoted, and stored at –80 °C.

### Statistical analysis

Statistical analysis of the quantum yield for different PP-IX nanostructures, FMN oxidation, and porphyrin metabolite accumulation were performed using GraphPad Prism software (GraphPad Software). Statistical comparisons were carried out using ordinary one-way ANOVA analysis and Tukey’s multiple comparison test or the unpaired *t*-test (two-tailed).

### Data availability

The mass spectrometry data can be accessed at MassIVE repository (https://massive.ucsd.edu/ProteoSAFe/static/massive.jsp, Project ID MSV000087196). All remaining data are contained within the article.

## Supporting information

This article contains supporting information.

## Conflict of interest

The authors declare that they have no conflicts of interest with the contents of this article.
