# Supplementary material for: Protein-aggregating ability of different protoporphyrin-IX nanostructures is dependent on their oxidation and protein-binding capacity
Source: J Biol Chem. 2021 May 21;297(1):100778. doi: 10.1016/j.jbc.2021.100778 (PMC8253973; doi:10.1016/j.jbc.2021.100778)
Supplement: Table S1 and S2; Figures S1–S8 [file mmc2.docx]

**Supporting Information**

**Protein aggregating ability of different protoporphyrin-IX nanostructures is dependent on their oxidation and protein binding capacity ***

Dhiman Maitra1,#, Benjamin M. Pinsky3, Amenah Soherawardy1, Haiyan Zheng1, Ruma Banerjee2,3, M. Bishr Omary1,3

1Center for Advanced Biotechnology and Medicine, Rutgers University, Piscataway, NJ 08854, 2Department of Biological Chemistry, 3University of Michigan Medical School, Ann Arbor, MI 48109

**Table of contents**

[**Table S1** S-2](#_Toc69385109)

[**Table S2** S-3](#_Toc69385110)

[**Figure S1** S-4](#_Toc69385111)

[**Figure S2** S-5](#_Toc69385113)

[**Figure S3** S-6](#_Toc69385115)

[**Figure S4** S-7](#_Toc69385117)

[**Figure S5** S-8](#_Toc69385119)

[**Figure S6** S-10](#_Toc69385121)

[**Table S3** S-11](#_Toc69385123)

[**Table S4** S-12](#_Toc69385124)

[**Figure S7** S-13](#_Toc69385125)

[**Figure S8** S-15](#_Toc69385127)

**Table S1:** Table showing p-values from ordinary one-way ANOVA analysis and Tukey’s multiple comparison test for data shown in Fig. 2A.

| **Tukey's multiple**  **comparisons test** | **Significant** | **Adjusted**  **P Value** |
| --- | --- | --- |
| **pH4.5 vs. pH7.4** | No | 0.9997 |
| **pH4.5 vs. pH9** | Yes | 0.0260 |
| **pH4.5 vs. pH7.4 + Emp** | Yes | <0.0001 |
| **pH7.4 vs. pH9** | Yes | 0.0294 |
| **pH7.4 vs. pH7.4 + Emp** | Yes | <0.0001 |
| **pH9 vs. pH7.4 + Emp** | Yes | <0.0001 |

**Table S2:** The table shows p-values from ordinary one-way ANOVA analysis and Tukey’s multiple comparison test for data shown in Fig. 2C.

| [PP-IX], 6.25 µM |  |  |
| --- | --- | --- |
| **Tukey's multiple**  **comparisons test** | **Significant** | **Adjusted P Value** |
| **pH4.5 vs. pH7.4** | No | >0.9999 |
| **pH4.5 vs. pH9** | Yes | 0.0462 |
| **pH4.5 vs. pH7.4 + Emp** | No | 0.0525 |
| **pH7.4 vs. pH9** | Yes | 0.0463 |
| **pH7.4 vs. pH7.4 + Emp** | No | 0.0526 |
| **pH9 vs. pH7.4 + Emp** | No | 0.9997 |
| [PP-IX], 12.5 µM |  |  |
| **Tukey's multiple comparisons test** | **Significant** | **Adjusted P Value** |
| **pH4.5 vs. pH7.4** | No | 0.3892 |
| **pH4.5 vs. pH9** | Yes | 0.0008 |
| **pH4.5 vs. pH7.4 + Emp** | Yes | 0.0004 |
| **pH7.4 vs. pH9** | Yes | 0.0057 |
| **pH7.4 vs. pH7.4 + Emp** | Yes | 0.0026 |
| **pH9 vs. pH7.4 + Emp** | No | 0.9047 |
| [PP-IX], 25 µM |  |  |
| **Tukey's multiple**  **comparisons test** | **Significant** | **Adjusted P Value** |
| **pH4.5 vs. pH7.4** | No | 0.8757 |
| **pH4.5 vs. pH9** | Yes | <0.0001 |
| **pH4.5 vs. pH7.4 + Emp** | Yes | <0.0001 |
| **pH7.4 vs. pH9** | Yes | <0.0001 |
| **pH7.4 vs. pH7.4 + Emp** | Yes | <0.0001 |
| **pH9 vs. pH7.4 + Emp** | No | 0.0998 |
| [PP-IX], 30 µM |  |  |
| **Tukey's multiple**  **comparisons test** | **Significant** | **Adjusted P Value** |
| **pH4.5 vs. pH7.4** | No | 0.9979 |
| **pH4.5 vs. pH9** | Yes | 0.0002 |
| **pH4.5 vs. pH7.4 + Emp** | Yes | <0.0001 |
| **pH7.4 vs. pH9** | Yes | 0.0001 |
| **pH7.4 vs. pH7.4 + Emp** | Yes | <0.0001 |
| **pH9 vs. pH7.4 + Emp** | Yes | 0.0131 |
| [PP-IX], 30 µM |  |  |
| **Tukey's multiple**  **comparisons test** | **Significant** | **Adjusted P Value** |
| **pH4.5 vs. pH7.4** | No | 0.2773 |
| **pH4.5 vs. pH9** | Yes | <0.0001 |
| **pH4.5 vs. pH7.4 + Emp** | Yes | <0.0001 |
| **pH7.4 vs. pH9** | Yes | <0.0001 |
| **pH7.4 vs. pH7.4 + Emp** | Yes | <0.0001 |
| **pH9 vs. pH7.4 + Emp** | Yes | 0.0003 |

# **Figure S1**


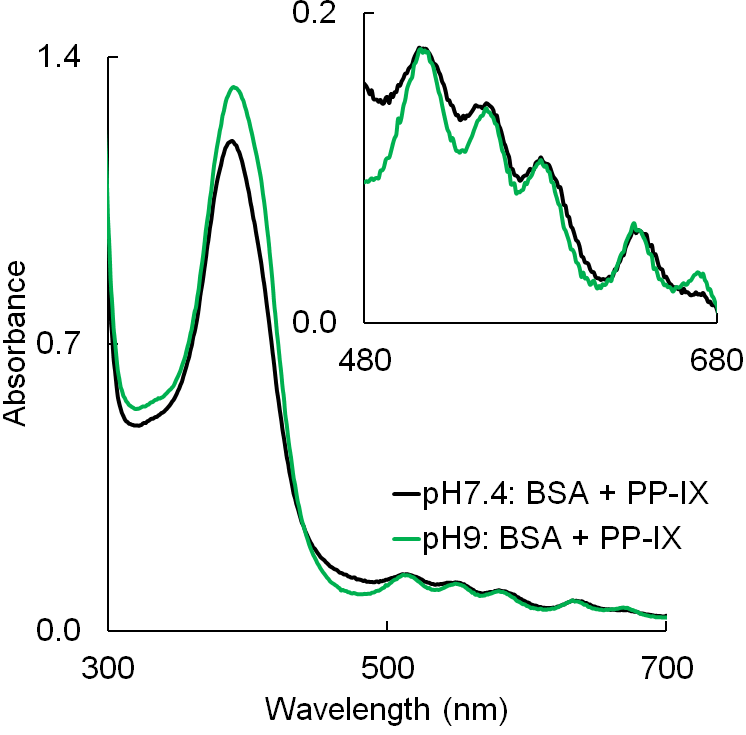


**Figure S1: BSA-PPIX complexes formed at pH7.4 and pH9 are indistinguishable.** Overlay of BSA+PP-IX absorbance spectra collected at pH7.4 and pH9 (from the experiment described in Fig.1). Inset shows the zoomed part of the spectra from 480-680 nm. Data is representative of three independent experiments.

# **Figure S2**


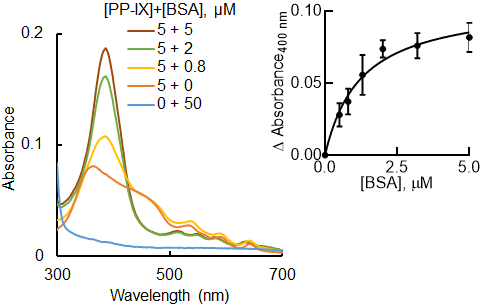


**Figure S2: PP-IX binding to BSA resolves PP-IX absorbance spectrum.** Absorbance spectra of different PP-IX + BSA mixtures (from the experiment described in Fig.5B, C). Inset shows the difference in absorbance of PP-IX (calculated from the relative increase in PP-IX absorbance after adding BSA) plotted as a function of BSA concentration. The data was fitted to a hyperbola (smooth line) using GraphPad Prism 8. Data are representative of three independent experiments, and inset show the average of three independent experiments ± standard deviation.

# **Figure S3**


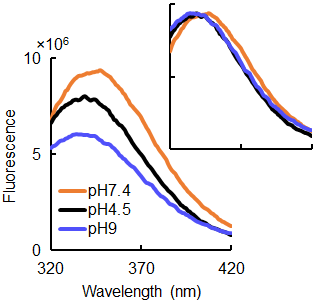


**Figure S3:** **Intrinsic fluorescence of BSA is dependent on the pH of the solution.** BSA (0.5 µM) at different pH, was excited at 280 nm, followed by collection of the fluorescence emission (320 to 420 nm). Inset shows the same spectra normalized to pH7.4 emission maxima. The data shown is representative of three independent experiments.

# **Figure S4**


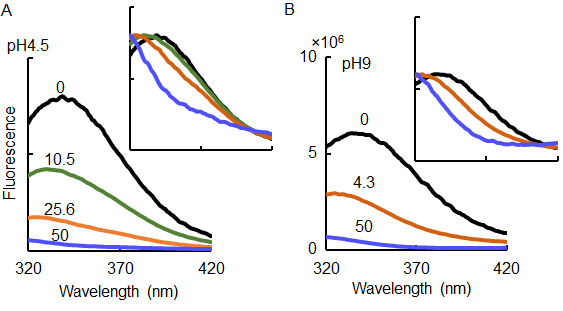


**Figure S4:** **PP-IX binding causes blue shift and quenching of BSA intrinsic fluorescence.** BSA (0.5 µM) was incubated with PP-IX (0-50 µM) for 30min at pH4.5 (panel A) and pH9 (panel B), and emission spectra were recorded after exciting the sample at 280 nm. The numbers above the spectral traces show PP-IX concentration in µM. Inset shows the same spectra normalized to the BSA-alone sample emission maxima. The data are representative of three independent experiments.

# **Figure S5**


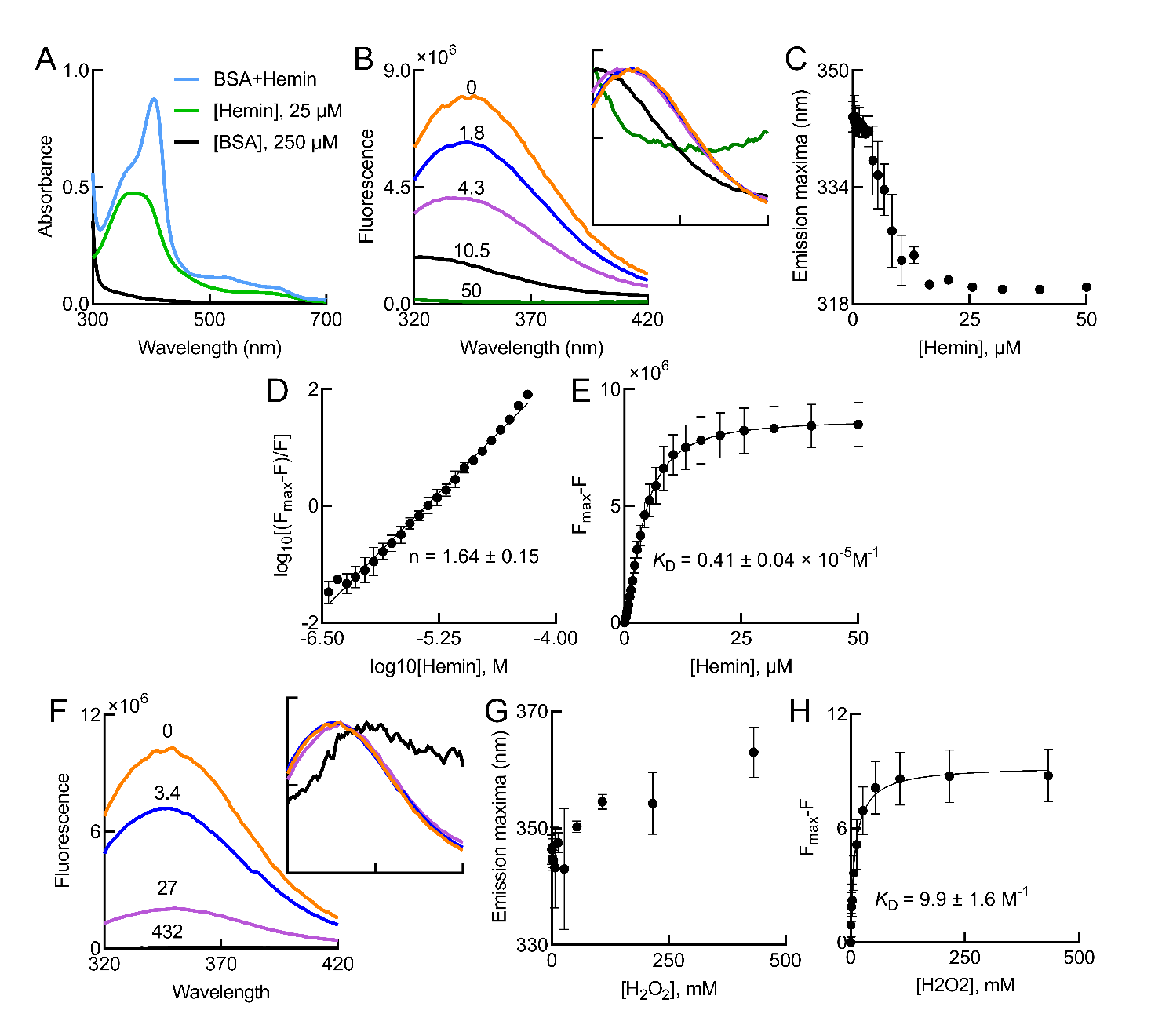


**Figure S5: Hemin and H_2_O_2_ quenches of BSA intrinsic fluorescence. A)** Hemin (25 µM) was incubated with BSA (250 µM) at pH7.4 for 30min and absorbance spectra were collected. Hemin showed a characteristic broad absorbance spectra indicative of higher order aggregates, which resolved into sharper Soret band upon BSA binding. **B-E)** BSA (0.5 µM) was incubated with hemin (0-50 µM) for 30min (pH7.4), then the emission spectra were recorded after exciting the sample at 280 nm (panel B). The numbers above the spectral traces represent hemin concentration in µM. Inset shows the same spectra normalized to the BSA-alone sample emission maxima. Changes in BSA emission maxima as a function of hemin concentration (panel C). Double log plot of log10[(Fmax-F)/F] versus log10[Hemin] (panel D). Plot of Fmax-F as a function of hemin concentration (panel E). The smooth line (in panel E) shows fitting of the data to a nonlinear regression model of saturation binding – ‘specific binding with Hill Slope’ equation in GraphPad Prism 8. **F-H)** BSA (0.5 µM) was incubated with H_2_O_2_ (0-500 µM) for 30min (pH7.4), and emission spectra were recorded after exciting the sample at 280 nm (panel F). The numbers above the spectral traces represent the H_2_O_2_ concentration in µM. Inset shows the same spectra normalized to BSA-alone sample emission maxima. Changes in BSA emission maxima as a function of hemin concentration (panel G). Plot of Fmax-F as a function of hemin concentration (panel E). Data shown in panels A, B and F are representative of three independent experiments. The data in panels C, D, G, H are average of three independent experiments ± standard deviation.

# **Figure S6**

**
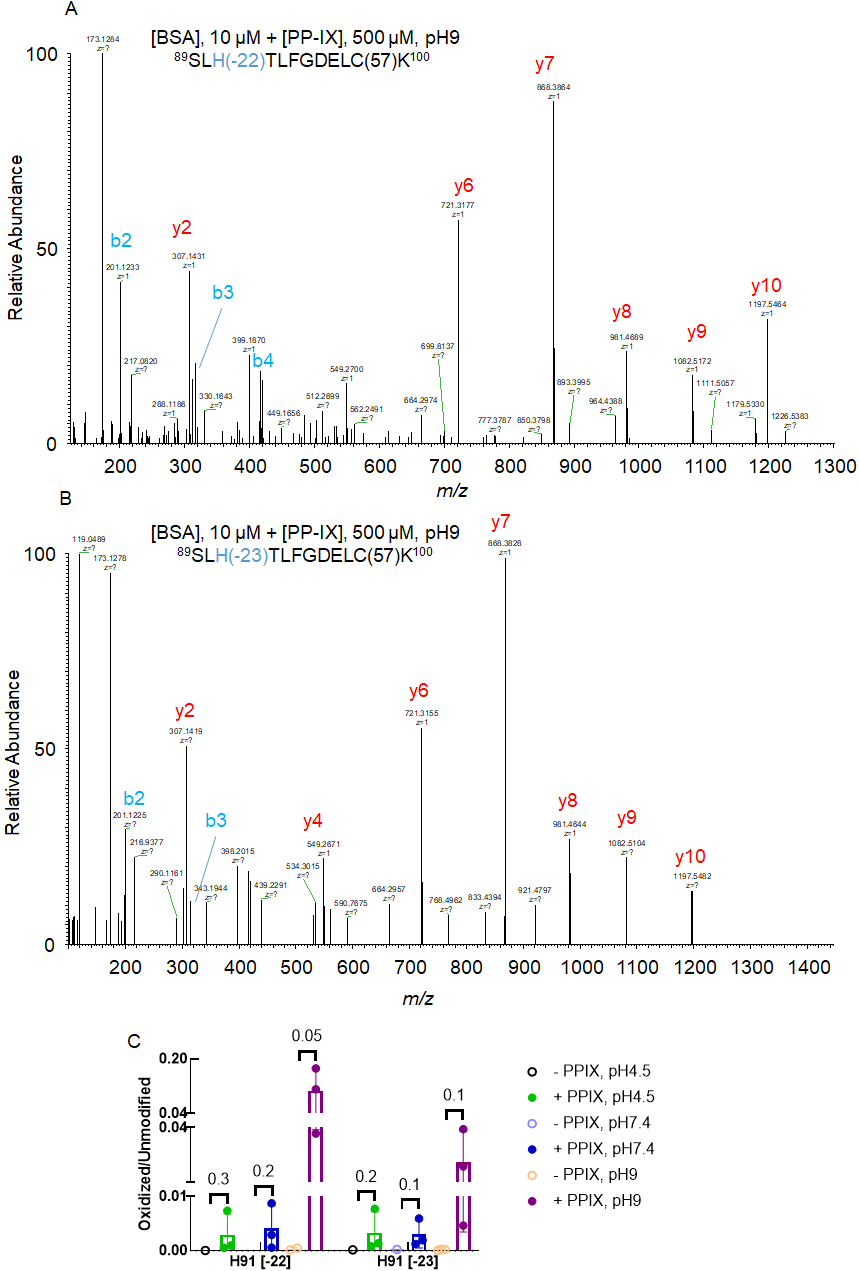
**

**Figure S6:** **Protoporphyrin-IX oxidizes BSA histidine-91.** BSA (10 µM) was treated with PP-IX (500 µM) for 30minu at the indicated pH. The reaction was quenched by extracting PP-IX followed by analysis of the protein pellet by LC-MS/MS, as detailed in Experimental Procedures. The amino acid residue (one letter abbreviation) and the type of oxidation (denoted as change in mass; see Supplementary Table S3 for details regarding the type of oxidation) is highlighted. **A, B)** Annotated representative LC-MS/MS spectra from the indicated reaction conditions. **C)** Ratio of the abundance (quantified by Skyline software) of the oxidized peptide to unmodified peptide. The data shown is an average of three independent experiments ± standard deviation. The p-values are included above the brackets and were calculated using the unpaired t-test (-PP-IX versus +PP-IX).

**Table S3:** List of potential oxidative modifications from the Unimod database that were searched for in control and PP-IX-treated BSA samples.

| **Unimod Accession #** | **Interim Name** | **Description** | **Modified amino acid** | **Δ**  **monoisotopic mass** |
| --- | --- | --- | --- | --- |
| 344 | Argglutamicsealde | Arginine oxidation to glutamic  semialdehyde | R | -43 |
| 1914 | Met->AspSA | Methionine oxidation to aspartic semialdehyde | M | -32 |
| 360 | Pyrrolidinone | Proline oxidation to  pyrrolidinone | P | -30 |
| 348 | His->Asn | His->Asn substitution | H | -23 |
| 349 | His->Asp | His->Asp substitution | H | -22 |
| 352 | Lysaminoadipicsealde | Lysine oxidation to aminoadipic semialdehyde | K | -1 |
| 351 | Kynurenin | Tryptophan oxidation to kynurenin | W | +4 |
| 359 | Pyroglutamic | Proline oxidation to  pyroglutamic acid | P | +14 |
| 288 | Oxolactone | Tryptophan oxidation to oxolactone | W | +14 |
| 35 | Hydroxylation | Oxidation or  Hydroxylation | MWY | +16 |
| 1922 | Pro->HAVA | Proline oxidation to 5-  hydroxy-2- aminovaleric acid | P | +18 |
| 350 | Hydroxykynurenin | Tryptophan oxidation  to hydroxykynurenin | W | +20 |
| 1923 | Delta:H(-4)O(2) | Tryptophan oxidation to beta-unsaturated- 2,4-bis- tryptophandione | W | +28 |
| 425 | Dihydroxy | Dihydroxy | MWY | +32 |
| 1384 | Homocysteic acid | Methionine oxidation to homocysteic acid | M | +34 |
| 345 | Cysteic acid | Cysteine oxidation to  cysteic acid | CWY | +48 |
| 1918 | Carbonyl | Aldehyde and ketone modifications | AEILQRSV | +14 |

# **Table S4: List of all peptide sequences identified, with the corresponding m/z, modifications, site of modification and peptide identification score for BSA and BSA+PP-IX reaction mixtures at pH4.5, 7.4 and 9, for three independent trials.**

# **Figure S7**


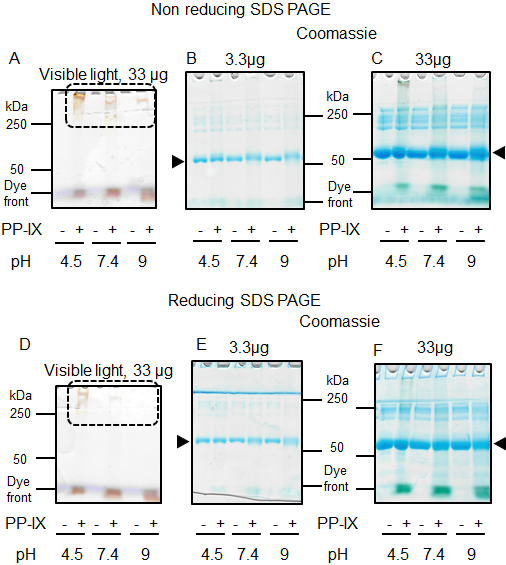


**Figure S7:** **PP-IX binding does not lead to BSA aggregation.** BSA (10 µM) was treated with PP- IX (500 µM) for 30min at the indicated pH. The incubation was quenched by adding non- reducing (panels A-C) or reducing (panels D-F) SDS-PAGE sample buffer. After SDS-PAGE, the gels were scanned under visible light (to visualize PP-IX, panels A and D), then stained with Coomassie (panels B, C, E, F) to visualize the proteins. The BSA monomer band is marked with an *arrowhead*. PP-IX migrated differently in a pH dependent fashion. Higher order PP-IX nanostructures (at pH 4.5) are more prominent at the top of the gel, particularly under non-reducing conditions (panels A,D). The band corresponding to the BSA monomer became more diffuse, in the presence of PP-IX, particularly as the pH increased (panels B and E, *arrowhead*). The data is representative of three independent experiments.

# **Figure S8**

**
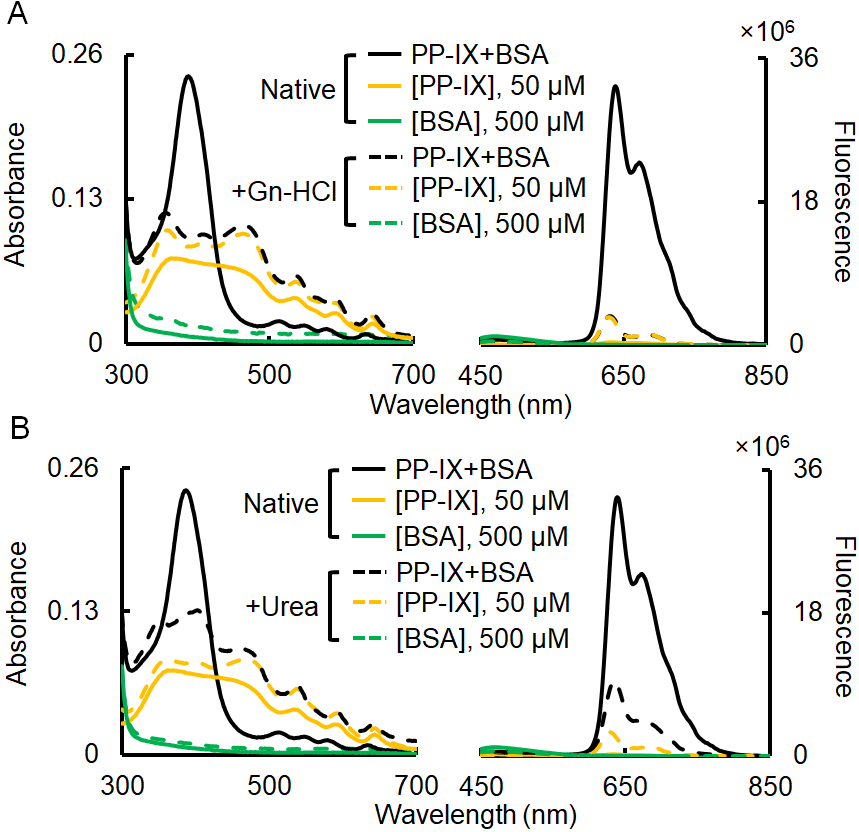
**

**Figure S8: Denatured BSA does not bind PP-IX.** The BSA solution was prepared either in guanidine (+Gn-HCl, panel A), or urea (+Urea, panel B), and treated with PP- IX and UV-Vis spectra of the samples were collected. Experiments described in Fig.8 and in the current figure were performed at the same time. The displayed absorbance and fluorescence spectra is overlayed with the ‘Native” samples (from the experiment described in Fig.8; collected at the same time) for comparison. Data is representative of three independent experiments.
